# Studies on the mechanism of ototoxic action of cisplatin and the antagonistic effect of polyphenolic compounds

**DOI:** 10.3389/fphar.2025.1586243

**Published:** 2025-04-25

**Authors:** Shengyu Han, Jinjun Sun, Wenpeng Li, Jihong Li, Haoming Yu, Shuai Wang, Yuhua Chi

**Affiliations:** ^1^ School of Clinical Medicine, Shandong Second Medical University, Weifang, Shandong, China; ^2^ Department of General Medicine, Affiliated Hospital of Shandong Second Medical University, Weifang, Shandong, China; ^3^ Department of Radiotherapy, Affiliated Hospital of Shandong Second Medical University, Weifang, Shandong, China

**Keywords:** antagonistic effect, polyphenols, cisplatin, signalling pathway, apoptosis

## Abstract

Cisplatin is a highly effective broad-spectrum anticancer drug, but its severe ototoxicity limits its clinical application. Cisplatin ototoxicity is mainly manifested as irreversible hearing loss, and its mechanism involves various pathways such as DNA damage, oxidative stress, inflammatory response, mitochondrial dysfunction, and ferroptosis. In recent years, natural polyphenols have shown great potential in combating cisplatin ototoxicity due to their powerful antioxidant, anti-inflammatory and anti-apoptotic properties.A variety of polyphenolic compounds, such as resveratrol, curcumin, quercetin, etc., can effectively attenuate the damage of cisplatin on Corti organs, spiral ganglion neurons and vascular striatum by scavenging free radicals, inhibiting the release of inflammatory factors, and regulating the expression of apoptosis-related proteins. In addition, some polyphenols can enhance the anti-tumour effect while antagonizing ototoxicity.Although polyphenols show good application prospects in the prevention and treatment of cisplatin ototoxicity, there are still some problems that need to be solved, such as the low bioavailability of polyphenols, the mechanism of action has not yet been fully elucidated, the optimal dosing regimen has not yet been determined, whether there is any superimposed effect of combining the various types of polyphenols, and whether the oral polyphenols can exert an otoprotective effect through the regulation of the intestinal flora through the intestinal-auricular axis.This study provides new insights into polyphenols as potential drug candidates for CIO by summarising the cytotoxic mechanisms of cisplatin and the mechanism of action of polyphenols targeting these mechanisms in order to retard the progression of CIO. It provides new ideas and approaches for the next step focusing on the development of highly effective and low-toxic polyphenols for clinical control of cisplatin ototoxicity.

## 1 Introduction

Cisplatin is a chemotherapeutic agent that is widely used as an antitumor agent for the treatment of various types of cancers, such as ovarian, prostate, testicular, lung, nasopharyngeal, esophageal, lymphoma, squamous cell carcinoma of the head and neck, and osteogenic sarcoma (Tang et al., 2024; Wang L. et al., 2023). However, its prevalent effects of renal damage, neurotoxicity and hearing impairment have severely limited the clinical use of cisplatin (Fetoni et al., 2022; Crona et al., 2017). Cisplatin-induced ototoxicity (CIO) is characterised by hair cell death through the formation of DNA adducts, mitochondrial dysfunction, oxidative stress and inflammation, ultimately leading to apoptosis, necrotic apoptosis, pyroptosis, or ferroptosis (Tan and Vlajkovic, 2023). The ototoxicity of cisplatin is permanent sensorineural hearing loss due to the major damage to the spiral apparatus (Corti organ), spiral ganglion neurons (SGN) and vascular striae (Rybak et al., 2019; Anfuso et al., 2022) and the lack of regenerative capacity of mammalian hair cells and SGN.

Given the key role of oxidative stress in CIO, antioxidants have been used as otoprotective agents in a large number of studies targeting the prevention of cisplatin-induced hearing loss, such as N-acetylcysteine, sodium thiosulfate, amphotericin, vitamins, statins, dexamethasone, D-methionine, and ginkgo biloba extract (Rose et al., 2024). However, most of the experimental tests have been conducted in animal models and *in vitro* experiments, and only a few clinical trials have been conducted. In these studies, polyphenolic compounds demonstrated potent antioxidant and anti-inflammatory properties.

Polyphenols are natural bioactive compounds, mostly from herbs, fruits, vegetables and medicinal plants. Recent studies strongly support the role of polyphenols in the prevention of diseases, especially aging, cancer, cardiovascular and neurodegenerative diseases (Xiang et al., 2023; Dalgaard et al., 2019; Grabska-Kobyłecka et al., 2023; Zhou et al., 2016). Polyphenols can prevent ototoxicity of cisplatin by improving cellular antioxidant homeostasis, modulating signaling pathways, mitigating inflammatory responses, and ameliorating the disruption of cisplatin’s intra-auricular environment in a dose- and time-dependent manner (Maiuolo et al., 2022).Moreover, after studies of natural compounds with anti-tumor properties, polyphenols have emerged as strong chemotherapeutic sensitization candidates (Jakobušić Brala et al., 2023; Abotaleb et al., 2020; Bouyahya et al., 2022; Ko et al., 2017), inhibiting the oncogenic transformation of normal cells through the regulation of relevant genes, tumor growth and development, angiogenesis and metastasis, downregulation of various oncogenic-related molecules and upregulation of tumor suppressor proteins, modulation of reactive oxygen species levels in cells to regulate cell proliferation, survival and apoptosis, and also transformation of the gut microbiota to acquire bioactivity-promoting properties to inhibit cancer development and progression (Anantharaju et al., 2016).Polyphenol combination therapy significantly improves anticancer efficacy and reduces drug resistance and chemotherapeutic toxicity through multi-target synergy. The application of nanocarriers can significantly improve the water solubility and stability of polyphenols, thus enhancing their selective killing ability on tumor cells (Vladu et al., 2022; Li et al., 2016).Therefore, the chemotherapy regimen of cisplatin combined with polyphenols has the potential to become a new option for cancer treatment in the future.

Current research in the field of ototoxicity protection is still dominated by experimental animal models, and in this paper, “polyphenols,” “flavonoids,” “anthocyanin,” “flavonols,” “flavones” “flavanones,” “flavan-3-ols,” “isoflavones,” “stilbenes,” “phenolic acids,” “lignans,” “cisplatin-induced ototoxicity” as search terms in PubMed, a total of 79 papers were retrieved and combined with de-duplication to screen 55 preclinical studies in which polyphenols were used as the primary intervention. We analyzed the protective mechanism of polyphenols against cisplatin ototoxicity in these studies, and before that, we comprehensively summarized and reviewed the cytotoxic mechanism of cisplatin. In this way, we hope to provide new insights into polyphenols as potential drug candidates for CIO.

## 2 Mechanism of cisplatin ototoxicity

Cisplatin enters the cochlea, mainly through the blood-strial barrier and the blood-perilymph barrier (Sung et al., 2024). After entering the cochlea, cisplatin is taken up into cells by diffusive and passive transport, copper transporter protein 1 (Ctr1) and organic cation transporter protein 2 (OCT2), endocytosis, and toxicity through DNA adduct formation, oxidative stress, mitochondrial dysfunction, inflammatory response, and ferroptosis (Elmorsy et al., 2024).

### 2.1 Cisplatin-induced DNA damage and apoptosis

**FIGURE 1:**
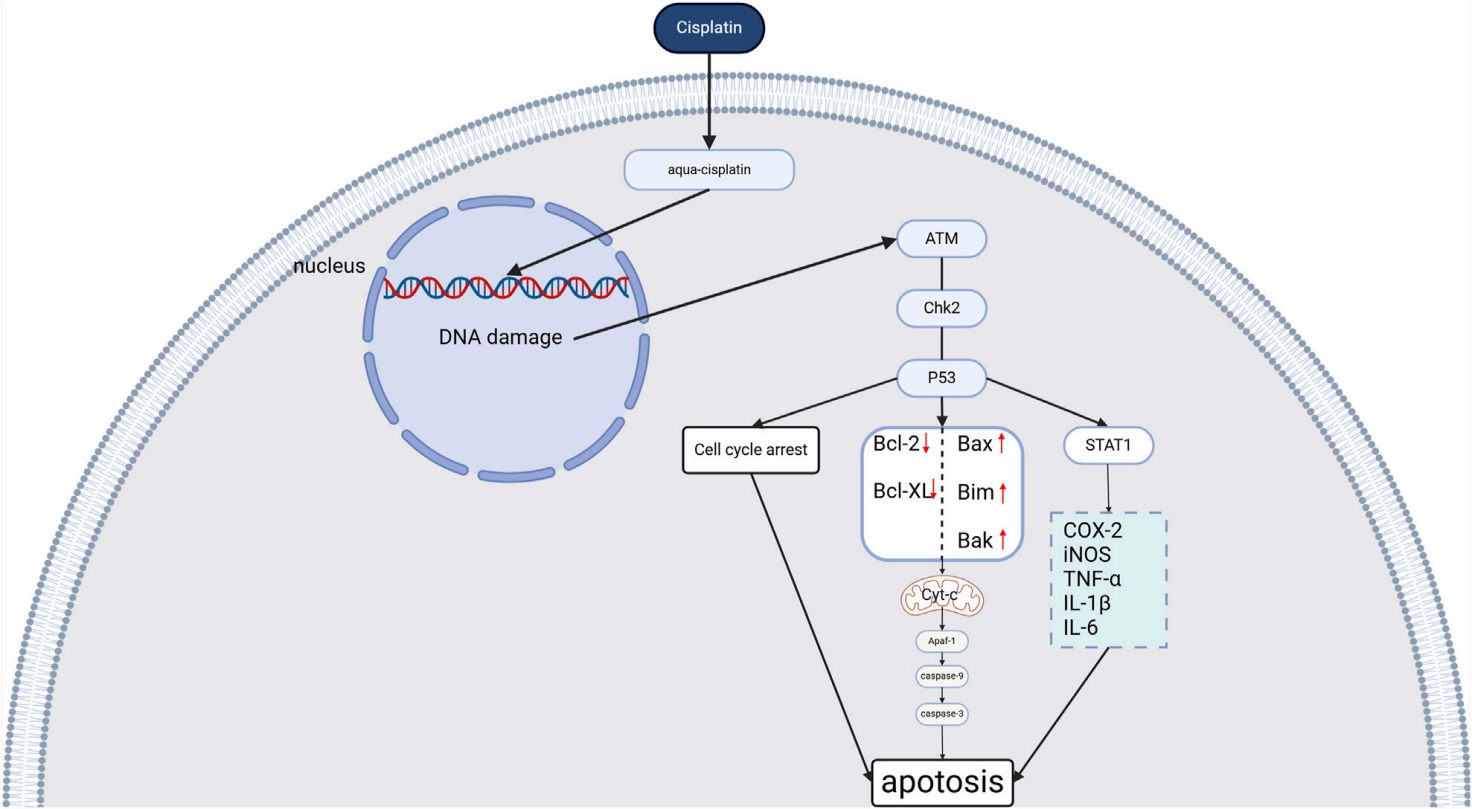
Cisplatin-induced cochlear DNA damage (Created in https://BioRender.com).

Once cisplatin enters the cell it can be hydrolyzed to form toxic aqua-cisplatin complexes and form intra-and interstrand crosslinks with DNA (Johnstone et al., 2016), the formation of such DNA adducts induces structural distortions of the DNA helix, which in turn leads to DNA damage, which activates multiple signaling pathways, one of which is the activation of the ATM-Chk2-p53 pathway (Benkafadar et al., 2017). Ataxia Telangiectasia Mutated (ATM) is a protein kinase that is activated in response to DNA double-strand breaks (DSBs). ATM can activate p53 either through activation of ATM checkpoint kinase 2 (Chk2) or directly. p53 activation activates the downstream Bcl family of proteins and the transcriptional activator of transcription 1 (STAT1). P53 activates the downstream Bcl family of pro-apoptotic proteins (Bax, Bak, Bim, etc.) and reduces the number of anti-apoptotic proteins (Bcl-xl, Bcl-2, etc.), which triggers the release of Cyt-c from the mitochondria, and ultimately activates the downstream cytosolic asparagine caspases that cause apoptosis (Cederroth et al., 2024). Activated STAT1 promotes the transcription of inflammatory genes such as COX-2, iNOS, and TNF-α, leading to an inflammatory response (Mukherjea et al., 2011). (See Figure 1)

### 2.2 Cisplatin induces oxidative stress

The cochlea’s high metabolic rate results in its production of abundant reactive oxygen species (ROS), so it requires an active antioxidant defense system [glutathione (GSH), oxidized glutathione (GSSG), and antioxidant enzymes (superoxide dismutase (SOD), catalase (CAT), glutathione peroxidase (GPx), and glutathione reductase (GR)] to achieve a dynamic oxidant-antioxidant balance to maintain healthy hearing. However, when cisplatin acts, it can induce oxidative stress by generating reactive oxygen species (ROS) and depleting cellular antioxidants.

Cisplatin is able to bind directly to the sulfhydryl groups of antioxidant enzymes in the cochlea, leading to the inactivation of the enzymes, and it also decreases the levels of glutathione and NADPH (Nicotinamide Adenine Dinucleotide Phosphate), both of which are essential for the activity of the enzyme glutathione peroxidase (Gündoğdu et al., 2019). Together, these changes lead to a decrease in antioxidant enzyme activity in the cochlea and an increase in the amount of ROS in the cells. Cisplatin activates NOX (nicotinamide adenine dinucleotide phosphate oxidase) (especially the NOX3 isoform) (Ramkumar et al., 2021), xanthine oxidase (XO) (Tan and Vlajkovic, 2023), and increases the production of superoxide radicals. In addition, mitochondrial dysfunction is another important mechanism leading to ROS production.

Reactive oxygen species (ROS) and their derived superoxide radicals can trigger cytotoxic effects through multiple mechanisms: first, excessive generation of ROS induces lipid peroxidation and leads to depletion of intracellular antioxidant enzyme levels, exacerbating the disruption of redox homeostasis; second, ROS induces the release of Cyt-c from the mitochondria, which in turn activates the caspase-9 and caspase-3 cascade reaction inducing apoptosis (Rybak et al., 2019); furthermore, ROS interacting with nitric oxide can form peroxynitrite (ONOO-), leading to protein inactivation; meanwhile, ROS generate reactive aldehydes such as 4-hydroxynonenal (4-HNE) through the formation of hydroxyl radicals and a series of reactions, which in turn trigger calcium ion endocytosis (Callejo et al., 2015).

However, signaling pathways that antagonize oxidative stress also exist in cells. Nrf2 is a major regulator of the cellular oxidative stress response and maintenance of redox homeostasis (Ma, 2013), and is regulated by signaling pathways such as NF-κB, Notch, PI3K-AKT, and Brca-1 (Li et al., 2023a), with the PI3K/AKT signaling pathway being a sensory hair cell important regulator for survival against oxidative stress (Kucharava et al., 2019). Activated Nrf2 promotes the gene expression of several antioxidant enzymes and proteins, such as superoxide dismutase (SOD), glutathione S-transferase (GST), glutathione peroxidase (GPx), heme oxygenase-1 (HO-1), and NAD(P)H as a means of protecting the cells from oxidative stress and maintaining the dynamic balance between oxidation and antioxidant protection. Activation of NF-κB also activates the downstream JNK pathway, leading to the production of ROS, which may cause further damage to the OHC (Wang X. et al., 2023).

### 2.3 Cisplatin induces mitochondrial dysfunction

When cisplatin enters the mitochondria, it acts directly on the inner mitochondrial membrane, leading to the impairment of the function of the electron transport chain, the reduction of ATP production and the induction of ROS generation (Elmorsy et al., 2024), and the inhibition of the efficient antioxidant defence system composed of glutathione (GSH) and enzymes, such as superoxide dismutase (SOD) and glutathione peroxidase (GPx), in the interstitial space of the mitochondrial membrane. Meanwhile, large amounts of ROS trigger mtDNA mutations when acting on mitochondrial DNA, protein oxidation when acting on respiratory chain proteins, and lipid peroxidation when acting on mitochondrial membranes (Tan and Song, 2023),these damages further lead to mitochondrial dysfunction, which in turn prompts more ROS production, forming a positive feedback loop (Tan and Vlajkovic, 2023).

**FIGURE 2:**
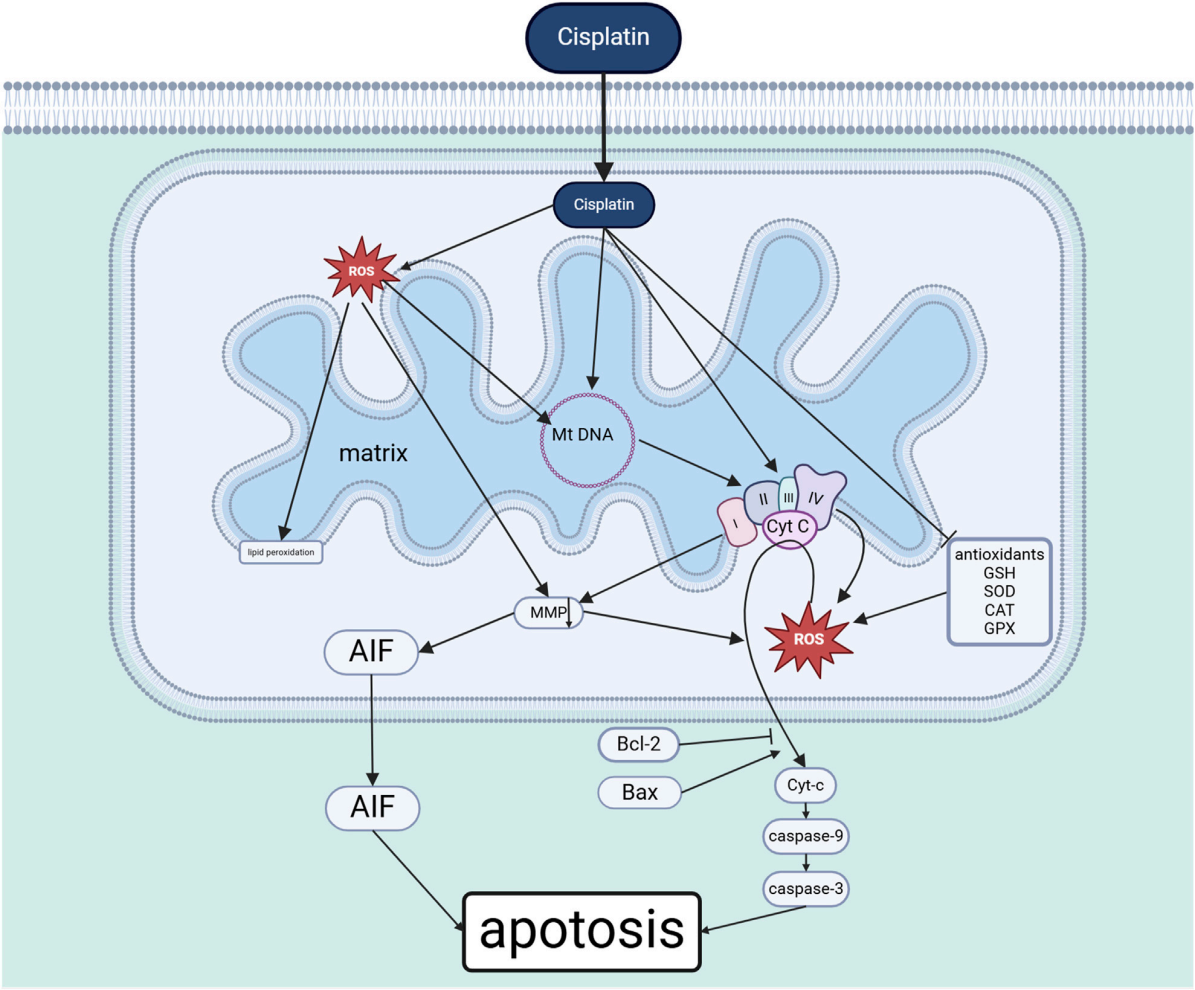
Cisplatin-induced cochlear mitochondrial dysfunction (Created in https://BioRender.com).

Dysfunctional mitochondria release into the cytoplasm, it binds to with Apaf-1, triggering caspase-9, caspase-3 related cascade reactions that lead to apoptosis (Santucci et al., 2019; Lu et al., 2022).Anti-apoptotic proteins such as Bcl-2 in the BCL-2 family and Bax and other pro-apoptotic members of the BCL-2 family balance and maintain normal mitochondrial function (Li et al., 2023b), and pro-apoptotic Bcl-2 family members induce the release of Cyt-c, which is inhibited by anti-apoptotic members. In addition, when mitochondria are damaged, apoptosis-inducing factor (AIF) is translocated from the mitochondria to the nucleus, where it may mediate chromatin condensation and large-scale DNA fragmentation by binding to DNA (Hong et al., 2004), leading to cell death (See Figure 2).

### 2.4 Cisplatin induces inflammation

The mechanism of cellular damage caused by cisplatin is inextricably linked to inflammation. The entry of cisplatin into the inner ear leads to the formation of inner ear cellular damage products (DAMPs), which, together with cisplatin, bind to toll-like receptors (TLRs) (Babolmorad et al., 2021) and activate downstream signaling, producing transcription factors (e.g., nuclear factor kappa B (NF-κB), activator protein-1 (AP-1), and interferon regulatory factor 3 (IRF3)), which in turn regulate the expression and secretion of proinflammatory signaling molecules. NF-κB induces cytokines such as tumor necrosis factor α (TNF-α), interleukin-1β (IL-1β), and interleukin-6 (IL-6) to bind to their respective receptors, which initiates and prolongs the progression of inflammation and contributes to the generation of ROS (Gong et al., 2020). Among them, TNF-α and IL-1β can activate NF-κB and thus promote the secretion of excess pro-inflammatory cytokines, forming a positive feedback and amplifying the inflammatory response cascade, which ultimately leads to more OHC damage (Wang L. et al., 2023).

**FIGURE 3:**
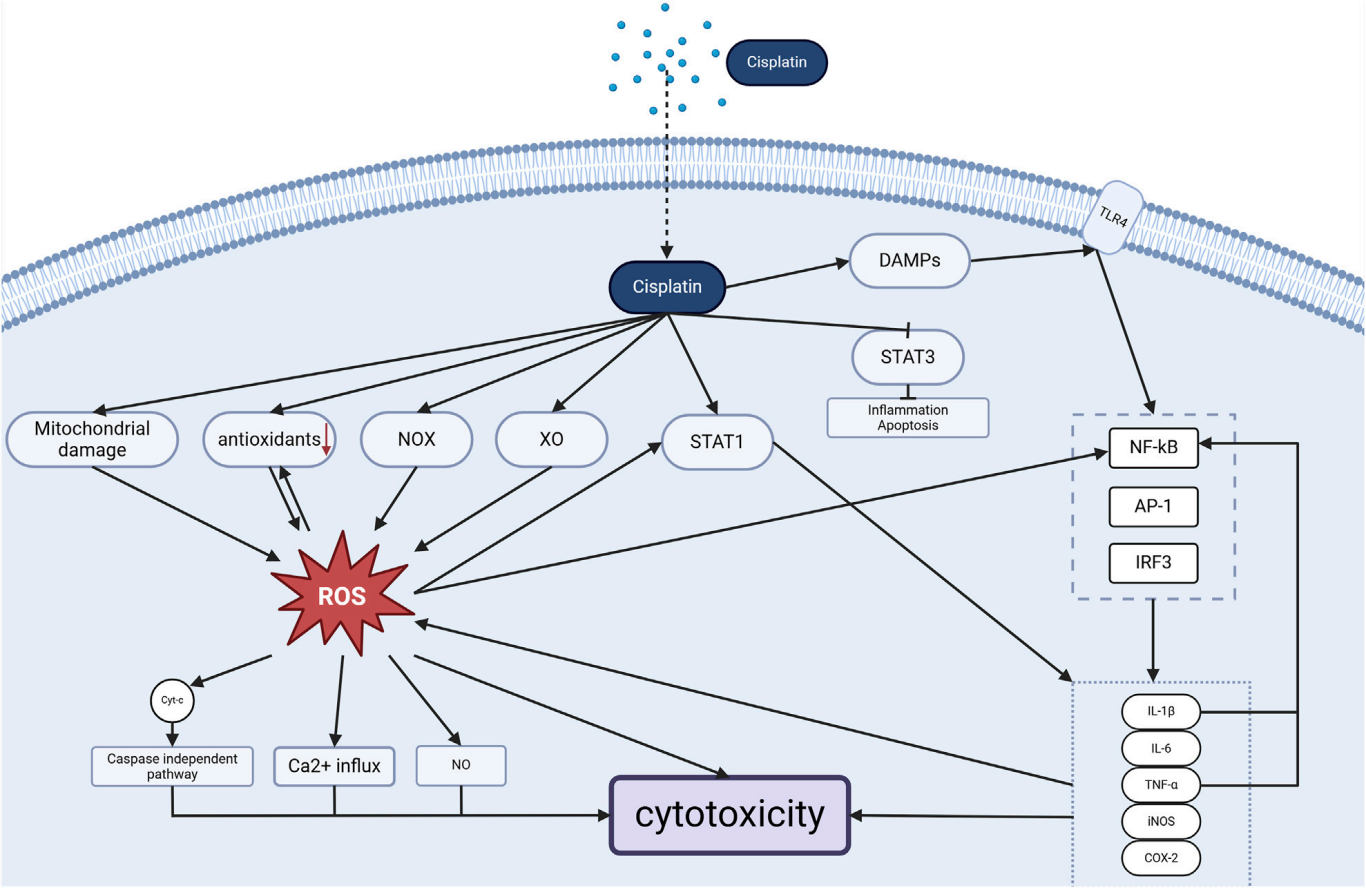
Cisplatin-induced cochlear Oxidative stress and inflammation (Created in https://BioRender.com).

Cisplatin also acts on the STAT family, inducing the protein expression of transcriptional activator of transcription-1 (STAT1) and down-regulating the expression of transcriptional activator of transcription-3 (STAT3) in the cochlea (Bhatta et al., 2019). STAT1 leads to the expression of various pro-inflammatory mediators, such as cyclooxygenase 2 (COX-2), iNOS, TNF-α, IL-1β, and IL-6, which further exacerbate the inflammation in the cochlea (Tan and Vlajkovic, 2023). However, STAT3 inhibits the cellular inflammatory response and promotes cell survival (Ramkumar et al., 2021). Increased ROS also activate STAT1 triggering the inflammatory process in the cochlea (Kaur et al., 2011), and the binding of inflammatory cytokines to their receptors further leads to an increase in ROS production in OHC cells.ROS and inflammatory responses reinforce each other, forming a positive feedback loop that further exacerbates OHC damage (See Figure 3).

### 2.5 Cisplatin induced iron death

**FIGURE 4:**
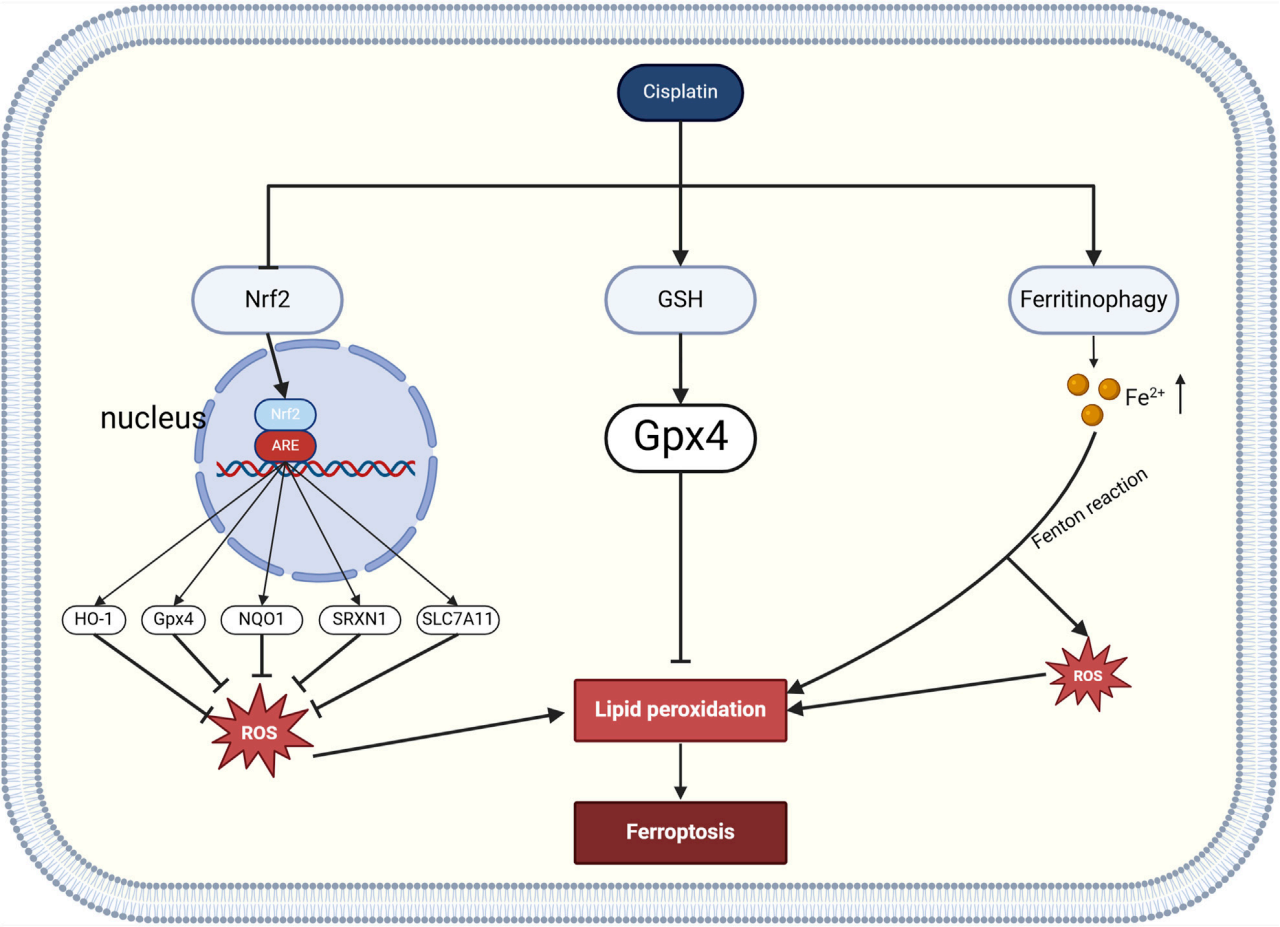
cisplatin-induced cochlear ferroptosis (Created in https://BioRender.com).

ferroptosis is a ROS-dependent form of cell death with two major biochemical features: iron accumulation and lipid peroxidation (Tang et al., 2021). Two major signaling pathways, nuclear factor erythroid 2-related factor 2 (NRF2) and glutathione peroxidase 4 (GPX4), play important roles in ferroptosis. Normally, the transcription factor Nrf2 and its inhibitory protein KEAP1 are tightly bound to inhibit activity, but in the presence of ROS, a conformational change in KEAP1 results in the release of NRF2, which binds to the antioxidant response element (ARE) and activates the downstream genes heme oxygenase-1 (HO-1), glutathione peroxidase 4 (GPX4), quinone oxidoreductase1(NQO1), thioredoxin reductase 1 (SRXN1), and transcription of solute carrier family 7 member 11 (SLC7A11), which enhance cellular antioxidant defenses and inhibit ferroptosis (Wang et al., 2022; Honkura et al., 2016). Cisplatin exacerbates hair cell damage through a dual mechanism: on the one hand, it activates nuclear receptor coactivator 4 (NCOA4)-mediated ferritinophagy, which prompts ferritin to release free Fe^2+^, generating a large amount of reactive oxygen species (ROS) via the Fenton reaction and inducing a lipid peroxidation chain reaction (Dai et al., 2024); on the other hand, inhibition of the antioxidant defense pathway of the transcription factor Nrf2 leads to reduced glutathione (GSH) synthesis and decreased activity of antioxidant enzymes (e.g., GPX4), further weakening cellular scavenging of free radicals (Lv et al., 2025). GPX4 was the first central inhibitor of ferroptosis to be discovered, and GPX4 uses glutathione to protect cells from ferroptosis through elimination of phospholipid peroxides to protect cells from ferroptosis (Stockwell et al., 2020) (Figure 4).

## 3 Polyphenols antagonise the mechanism of cisplatin ototoxicity

**FIGURE 5:**
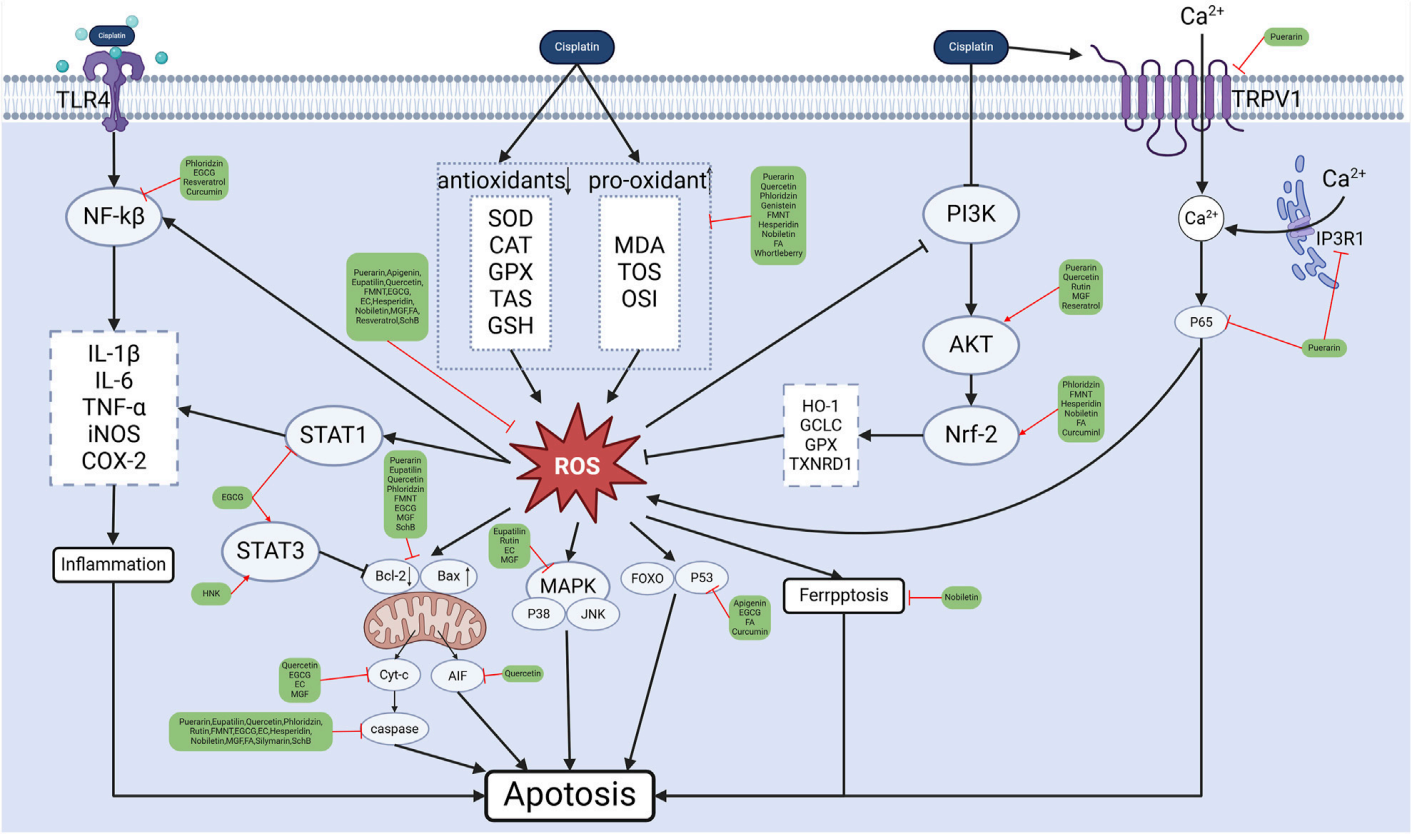
Polyphenols’ protective mechanism against ototoxicity. (Created in https://BioRender.com).

Polyphenols are a large group of natural products, the most common natural antioxidants, characterised by the presence of multiple phenolic units in their structure. There are approximately 8,000 known polyphenol compounds, of which more than 4,000 belong to flavonoids (Cheynier, 2005). In this context, the present study systematically combed natural polyphenols (both flavonoids and non-flavonoids) with ototoxic antagonistic effects in preclinical studies and reviewed the possible mechanisms of prevention of ototoxicity by these natural compounds (Figure 5 and Table 1).

**TABLE 1:** Protective effect of polyphenols against cisplatin-induced ototoxicity.

Name	Source	Research object	Effect↑	Effect↓	Ref
Puerarin	Chinese medical herb Radix Pueraria	HEI-OC1 cells,C57BL/6 mice	AKT,Cell viability	Bax,caspase-3,ROS,hair cell apoptosis	Xu et al. (2022)
		HEI-OC1 cells, male BALB/c mice	SOD,CAT,GPX	ABR thresholds, TRPV1,IP3R1,NF-κB,p65,Ca2+ overload,ROS,MDA,hair cell apoptosis	Lin et al. (2024)
Apigenin	Vegetables, fruits,herbs,plant-based beverages	Wild-type (AB) and Tg (Brn3c:mGFP) transgenic zebrafish		ROS,p53,FoxO, gadd45ba,serpine1,hair cell apoptosis, Ferroptosis	Kong et al. (2024)
Eupatilin	Artemisia	HEI-OC1 cells,The trans genic zebrafish line Tg (Brn3C:EGFP),C57BL/6J mice	Bcl-2	ROS,p38,JNK,Bax,caspase-3,PARP, hair cell apoptosis	Lu et al. (2022)
Quercetin	fruits and vegetables	female Wistar albino rats	DPOAE	Damage to the stria vascularis, organ of Corti and spiral ganglion	Gündoğdu et al. (2019)
		Transgenic zebrafish (Brn3C:EGFP)		hair cell apoptosis, Damage to the ultrastructure of hair cells	Lee et al. (2015a)
		male C57BL/6J mice	Bcl-2,MMPs,SOD,PI3K,Akt,TEER,VE-cad,ZO-1	ABR thresholds,caspase-3,Bax,MDA,ROS,Cyt-c,AIF	Huang et al. (2024)
Phloridzin	apples and pears	Balb/C female mice	SOD,GSH	MDA,TNF-α,IL1β,NF-Kβ	Un et al. (2021)
		HEI-OC1 cells	HO-1,Nrf2, JNK,ERK,p38,MAPK, Cell viability, Bcl-2	Bax,caspase-8,caspase-9,caspase-3	Choi et al. (2011)
Rutin	Vitamin P	C57BL/6 mice	PI3K,AKT	JNK,p38,MAPK,caspase-3,hair cell apoptosis, SGNs apoptosis	Zheng et al. (2022)
Genistein	Soybeans	female Sprague-Dawley rats	DPOAE,SOD,CAT,GPX,TAS	TOS,OSI,MDA	Tan et al. (2022)
Formononetin (FMNT)	herbal medicines (Angelica sinensis and Astragalus membranaceus)	C57BL/6 mice	PI3K,AKT,Nrf2,Gclc,Gpx2,Txnrd1,HO-1,Bcl–2	caspase-3,Bax,hair cell apoptosis,ROS,MDA,GSSG	Li et al. (2023a)
Epigallocatechin-3-gallate	green tea	HEI-OC1 cells	Cell viability	caspase-3,ROS,cell apoptosis	Cho et al. (2014a)
		HEI-OC1 cells,Sprague–Dawley rats	Cell viability,Bcl-2,MMPs	NF-κB,Cyt-c,caspase-3,NO,ROS,caspase-1, IL-1β	Kim et al. (2012)
		Kunming mice	Cell viability	MnSOD gene expression, Apoptosis of Spiral Ganglion Cells (SGCs)apoptosis	Xie et al. (2004)
		Swiss Webster mice		STAT1	Schmitt et al. (2009)
		UB/OC-1 cells, male Wistar rats, Severe Combined Immunodeficiency mice	Bcl–xL,STAT3,STAT3/STAT1	p53,caspase–3,Bax,LDH,ROS,ERK,STAT1,ABR thresholds,TNFα,COX2,iNOS,NOX3,Loss of OHCs	Borse et al. (2017)
Epicatechin	green tea	HEI-OC1 cells, Zebrafish	Cell viability,MMP	caspase-3,ROS,hair cell apoptosis, loss of kinocilium and stereocilia	Kim et al. (2008)
		HEI-OC1 cells, female Sprague–Dawley rats		ABR thresholds, MAPK,ROS,Cyt-c,caspase-3,Cell cycle arrest, Damage to OHCs, IHCs	Lee et al. (2010)
Hesperidin/Hesperetin	citrus fruit peels	wistar albino rats	TAS	MDA,TOS,OSI,MPO	Kara et al. (2016)
		Sfemale albino guinea pigs	SNR		Başoğlu et al. (2012)
		male C57BL/6J mice,HEI-OC1 cells	Nrf2,NQO1	ABR thresholds,caspase-3,PARP, hair cell apoptosis,ROS	Lou et al. (2024)
Nobiletin	citrus fruit peels	C57BL/6 mice,HEI-OC1 cells	Cell viability,SOD,GPX4,SLC7A11,FTH,FTL,GSH,NRF2,HO-1,NQO1,LC3B,SQSTM1,p62	Ferroptosis,caspase-3,ROS	Song et al. (2024)
Mangiferin	genus mango (Anacardiaceae)	HEI-OC1 cells,C57BL/6J mice,The Tg (Brn3C:EGFP) transgenic zebrafish	Cell viability,MMP,Akt,Map3k4,Map2k5,Prkca,Bcl-2	ROS,Bak,caspase-3,PARP,Cyt-c,ABR thresholds, hair cell apoptosis,JNK,P38,MAPK	Lu et al. (2024)
Pycnogenol	a French maritime pine bark extract	Male rats	DPOAE	Apoptosis	Eryilmaz et al. (2016)
silymarin	the seeds of Silybum marianum	HEI-OC1 cells	Cell viability	Apoptosis,caspase-3,PARP, Cell cycle arrest	(Cho et al., 2014b)
ferulic acid	vegetables, fruits and some beverages	HEI-OC1 cells, C57BL/6 mice	Gclc,GPX,CAT,SOD2,Nrf-2	ROS,caspase-3,PARP, hair cell apoptosis	Jo et al. (2019)
		Male adult Wistar rats	Nrf–2,HO–1	ABR thresholds,p5	Paciello et al. (2020)
resveratrol	grapes, berries, mulberries and peanuts	male albino guinea pigs		ABR thresholds,ROS,hair cell apoptosis	Yumusakhuylu et al. (2012)
		Albino–Wistar rats	DPOAE	ABR thresholds	Simsek et al. (2019)
		HEI-OC1 cells	Cell viability	ROS	Lee et al. (2015b)
		female Sprague-Dawley rats	CYP1A1	ABR thresholds, Damage to OHCs,NF-κB,IL1β,IL6,AhR,RAGE	Lee et al. (2020)
		HEI-OC1 cells, male C57BL/6J mice	DNMT1,miR-455-5p,Cell viability	GAS5,PTEN,ROS,ABR thresholds, hair cell apoptosis	Liu et al. (2021)
		HEI-OC1 cells, male C57BL/6J mice	Cell viability,miR-455-5p,PI3K,Akt	ROS,CAT,PTEN,ABR thresholds, hair cell apoptosis	Wu et al. (2024)
Schisandrin B	Schisandra chinensis	HEI-OC1 cells, male C57BL/6 mice	Bcl-2,DPOAE	Caspase-3,Bax,ROS,ABR thresholds, hair cell apoptosis	Li et al. (2024)
Curcumin	the plant Curcuma longa	male *Rattus norvegicus* Wistar rats	SNR		Arwanda et al. (2023)
		Male adult Wistar rats	Nrf,HO-1	ROS	Fetoni et al. (2014)
		Male adult Wistar rats	Nrf–2,HO-1	ABR thresholds,NF–κB,p53	Paciello et al. (2020)
		Male adult Wistar rats	DPOAE,Nrf – 2,HO-1	ABR thresholds, hair cell apoptosis	Fetoni et al. (2015)
Honokiol	magnolia bark	HEI-OC1 cells, Adult C57BL/6 mice	DPOAE, SIRT3	ABR thresholds, Damage to OHCs	Tan et al. (2020)
Ecklonia Cava Polyphenol	brown algae living in the seas of the Far East	HEI-OC1 cells	Cell viability		Düzenli et al. (2016)
caffeic acid	honey bee propolis	HEI-OC1 cells	Cell viability	ROS,caspase-3,caspase-8,Apoptosis	Choi et al. (2014)
whortleberry extract	whortleberry	adult male Wistar albino rats	DPOAE,TAS	TOS,OSI,cellular degeneration	Özdemir et al. (2019)
pomegranate extract		adult Wistar rats	DPOAE,SNR,TAS	TOS,OSI	Yazici et al. (2012)

Note: Akt:Protein Kinase B,PKB; Bax:Bcl-2, Associated X Protein; caspase-3:Cysteine-aspartic Protease 3; SOD:Superoxide Dismutase; CAT:Catalase; GPX:Glutathione Peroxidase; ABR:Auditory brainstem response; TRPV1:Transient Receptor Potential Vanilloid 1; IP3R1:Inositol 1,4,5 - Trisphosphate Receptor 1; NF–κB:Nuclear Factor–κB; MDA:Malondialdehyde; FoxO:Forkhead Box O; gadd45ba:Growth Arrest and DNA, Damage - Inducible Protein 45 Beta a; serpine1:Plasminogen Activator Inhibitor 1; JNK:c-Jun N-terminal Kinase; PARP:Poly (ADP, ribose) polymerase; DPOAE:the distortion product otoacoustic emissions; MMPs:Mitochondrial Membrane Potentials; PI3K:Phosphatidylinositol 3-Kinase; TEER:Transepithelial Electrical Resistance; VE-cad:Vascular Endothelial Cadherin; ZO-1:Zona Occludens-1; Cyt-c:Cytochrome c; AIF:Apoptosis-Inducing Factor; HO-1:Heme Oxygenase-1; Nrf2:Nuclear Factor-E2-Related Factor 2; ERK:Extracellular Signal-Regulated Kinase; MAPK:Mitogen-Activated Protein Kinase; caspase-8:Cysteine-aspartic Protease 8; caspase-9:Cysteine-aspartic Protease 9; SGNs:Spiral Ganglion Neurons; TAS:Total Antioxidant Status; OSI:Oxidative Stress Index; TOS:Total Oxidant Status; CLC:glutamate - cysteine ligase catalytic subunit; Gpx2:glutathione peroxidase 2; Txnrd1:thioredoxin reductase 1; SGCs:Spiral Ganglion Cells; MnSOD:Manganese Superoxide Dismutase; IL-1β:Interleukin-1β; STAT1:Signal transducer and activator of transcription 1; TNFα:Tumor Necrosis Factor Alpha; COX2:Cyclooxygenase-2; iNOS:Inducible Nitric Oxide Synthase; NOX3:NADPH, Oxidase 3; OHCs:outer hair cells; IHCs:inner hair cells; SNR:signal-to-noise ratio; NQO1:NAD (P) H quinone oxidoreductase 1; SLC7A11:Solute Carrier Family 7 Member 11; FTH:Ferritin Heavy Chain; FTL:Ferritin Light Chain; SQSTM1:Sequestosome 1; Map3k4:Mitogen-Activated Protein Kinase Kinase Kinase 4; Map2k5:Mitogen-Activated Protein Kinase Kinase 5; Prkca:Protein Kinase C Alpha; Gclc:Glutamate-Cysteine Ligase Catalytic Subunit; IL6:Interleukin 6; CYP1A1:Cytochrome P450 Family 1 Subfamily A Member 1; AhR:Aryl Hydrocarbon Receptor; RAGE:Receptor for Advanced Glycation Endproducts; DNMT1:DNA, methyltransferase 1; GAS5:Growth Arrest - Specific 5; PTEN:Phosphatase and Tensin Homolog; miR - 455, 5p:MicroRNA - 455, 5p; SV:Stria Vascularis; SGC:spiral ganglion cells.

Clinical evidence for polyphenolic natural products in the field of hearing protection is still limited, and Ginkgo biloba Linn (Ginkgoaceae) leaves extract (GBE) is one of the more well-studied polyphenolic agents, with active ingredients including Ginkgo flavonoids, Quercetin, Ginkgolides, Organic acids, and other components. In a randomized, double-blind, prospective clinical trial of 15 cancer patients, the GBE intervention significantly protected the mean distortion product otoacoustic emissions (DPOAEs) of the patients. DPOAEs) mean amplitude and signal-to-noise ratio (Dias et al., 2015). Notably, two systematic reviews and Meta-analyses further validated the clinical value of GBE-assisted treatment for sudden sensorineural hearing loss (SSNHL), showing that the combined intervention significantly increased the clinical recovery rate and improved the pure tone average (Si et al., 2022; Yuan et al., 2023). Doluperine capsules, a novel combination agent, demonstrated excellent hearing protection in patients with type 2 diabetes mellitus combined with SSNHL by targeting inflammatory and oxidative stress pathways, and its core ingredient curcumin, the most bioavailable polyphenol in the turmeric plant, may provide therapeutic benefits through a multi-targeted mechanism of action (Tajdini et al., 2023).

### 3.1 Flavonoids

Flavonoids have a general structural backbone of C6-C3-C6 in which two C6 units (ring A and ring B) are phenolic in nature. Due to variations in hydroxylation pattern and chromophore ring (ring C), they can be further classified into different subgroups such as flavonoids, flavanones, isoflavonoids, flavanols, flavan-3-ols and anthocyanins. Flavonoids ameliorate ototoxic drug-induced hearing impairment by acting as ROS scavengers or inhibitors of JNK, MAPK, and other apoptotic signaling pathways, and by inhibiting aldose reductase activity, xanthine oxidase (Liao et al., 2024), common natural flavonoid mechanisms of action are described below.

#### 3.1.1 Puerarin

Puerarin, one of the major components of Pueraria lobata, has been reported to have a wide range of pharmacological activities, including anti-inflammatory, antioxidant and anti-apoptotic effects. Puerarin reduces oxidative stress, lowers MDA levels, inhibits lipid peroxidation, protects cell membrane integrity, and inhibits cisplatin-induced damage to HEI-OC1 auditory cells by neutralizing superoxide anion, hydroxyl radical, hydrogen peroxide, and DPPH radicals (Yu et al., 2010). Puerarin can also inhibit cisplatin-induced hair cell injury by modulating apoptosis-related proteins (e.g., Bax and cleaved caspase-3) to attenuate the mitochondrial apoptotic pathway, reduce ROS accumulation and activate the Akt signaling pathway (Xu et al., 2022). Another study showed that Puerarin could block cisplatin-induced activation of TRPV1 and IP3R1, prevent intracellular calcium overload, and inhibit p65 to reduce excessive ROS production, thereby ameliorating cisplatin-induced ototoxicity and blocking apoptosis (Lin et al., 2024).

#### 3.1.2 Apigenin

Apigenin is a natural flavonoid found in a variety of foods and beverages with antioxidant, anti-inflammatory and anti-tumour properties. Apigenin inhibits cisplatin-induced mitochondrial ROS accumulation and protects HC from cisplatin-induced damage by inhibiting apoptosis-related signalling pathways such as p53 and FoxO (Kong et al., 2024).

#### 3.1.3 Eupatilin

Eupatilin is a pharmacologically active flavonoid found mainly in the genus Artemisia.Eupatilin reduces intracellular and mitochondrial ROS levels, inhibits the p38/JNK pathway, and reduces cisplatin-induced apoptosis of HEI-OC1 cells; it reduces cisplatin-induced hair cell loss, and protects auditory hair cells (Lu et al., 2022).

#### 3.1.4 Quercetin (QU)

QU is a widely used natural flavonoid compound that can be an effective antioxidant against cisplatin ototoxicity.QU scavenges a variety of free radicals such as peroxides and superoxides, as well as inhibits xanthine oxidase, blocks lipid peroxidation, chelates transition metals, and reduces cytosolic calcium uptake (Gündoğdu et al., 2019). It has been demonstrated that QU increases the number of hair cells, protects mitochondria, reduces apoptosis and maintains hair cell ultrastructure (Lee S. H. et al., 2015). Moreover, QU can activate the PI3K/AKT signalling pathway, inhibit cisplatin-induced oxidative stress, protect mitochondrial function, reduce apoptosis of the mitochondrial pathway in pericytes, maintain the integrity of the endothelial barrier, and attenuate hearing loss (Huang et al., 2024).

#### 3.1.5 Phloridzin and rhizodendron

Phloridzin and rhizoposide are the main flavonoid structural compounds of apple with antioxidant and anticancer effects (Un et al., 2021). Phloridzin inhibits mitochondrial dysfunction and caspase activation through activation of HO-1 expression induced by the Nrf2 and JNK pathway nuclei; and inhibits cisplatin-induced apoptosis in HEI-OC1 cells (Choi et al., 2011).

#### 3.1.6 Rutin

Rutin belongs to the vitamin P family and is a natural flavonol glycoside. Rutin protects hair cells and spiral ganglion neurons from cisplatin-induced ototoxicity by reducing ROS production after cisplatin exposure, activating PI3K/AKT signalling and inhibiting the JNK/p38/MAPK signalling pathway (Zheng et al., 2022).

#### 3.1.7 Genistein (genistein/strongylisoflavone, GST)

GST is a phytoestrogen found in large quantities in soybeans, GST increases the levels of antioxidant enzymes and decreases the levels of oxidant parameters, preventing cisplatin ototoxicity (Tan et al., 2022).

#### 3.1.8 Formononetin (FMNT)

FMNT is a natural flavonoid found in large quantities in herbs such as Angelica sinensis and Astragalus membranaceus.FMNT reduces ROS overload by activating the PI3K/AKT-Nrf2 signalling pathway and its downstream antioxidant genes, decreases the c-caspase-3/caspase-3 ratio (Li et al., 2023a) and inhibits apoptosis ameliorating cisplatin-induced hair cell death.

#### 3.1.9 Epigallocatechin-3-gallate (EGCG) and epicatechin

EGCG, a polyphenol abundant in green tea extract, is a STAT1 inhibitor.EGCG prevents cisplatin cytotoxicity through anti-apoptotic and antioxidant effects (Cho et al., 2014a); counteracts NO-induced ototoxicity by effectively inhibiting the activation of caspase-3, NF-κB, and preventing the destruction of hair cells in the organ of Corti, both *in vitro* and *ex vivo* (Kim et al., 2012); and protects auditory neurons from oxidative damage induced by H2O2 attack (Xie et al., 2004).EGCG acts through phosphatidylinositol 3-kinase (PI3K)/Akt signalling in cochlear NSCs to promote cell growth and neuronal differentiation, which can be used for the treatment of hearing loss (Zhang et al., 2016), and has an important protective mechanism against cochlear oxidative damage. Failing to provide additional protection against cisplatin toxicity after knockdown of the STAT1 gene in mice (Schmitt et al., 2009); EGCG reduced cisplatin-induced ROS generation and ERK1/2 and STAT1 activity, but retained STAT3 and Bcl-xL activity, allowing cisplatin to continue its anti-tumour effects (Borse et al., 2017). These studies suggest that EGCG is an ideal otoprotective agent for the treatment of cisplatin-induced hearing loss and may not compromise its antitumour efficacy.

Epicatechin (EC) is a minor component of green tea. It prevents cisplatin-induced ototoxicity by blocking ROS production and inhibiting changes in mitochondrial membrane potential (MMP) (Kim et al., 2008). EC inhibits cisplatin activation of JNK, ERK, Cyt-c and caspase-3 (Lee et al., 2010).

#### 3.1.10 Hesperidin and nobiletin

Hesperidin and hesperetin are natural flavonoid compounds found mainly in the peel of citrus fruits. Hesperidin is a potent natural antioxidant that increases antioxidant enzymes and reduces oxidants to prevent ototoxicity (Kara et al., 2016; Başoğlu et al., 2012). In addition, Hesperidin activates the Nrf2/NQO1 pathway, decreases ROS levels, and enhances the antioxidant capacity of hair cells and HEI-OC1 cells, thereby attenuating cisplatin-induced oxidative damage (Lou et al., 2024). Nobiletin is a polymethoxyflavonoid with antioxidant and anti-apoptotic properties. The protective function of nobiletin against cisplatin-induced ototoxicity has been attributed to the activation of autophagy and the activation of NRF2/GPX4, which in turn inhibits the onset of ferroptosis (Song et al., 2024).

#### 3.1.11 Mangiferin (MGF) and pycnogenol

Extracted from the genus Mangiferin, MGF protects against cisplatin-associated ototoxicity by down-regulating ROS accumulation, restoring mitochondrial function, and inhibiting apoptosis in in vitro (HEI-OC1 cells and cochlear hair cells) and *in vivo* (zebrafish larvae and C57BL/6 J mice) models and, the molecular mechanism of which may be attributed to its inhibition of the ROS-MAPK-caspase-3 signalling pathway (Lu et al., 2024). Pycnogenol prevents cisplatin-induced cochlear apoptosis and is protective against cisplatin ototoxicity (Eryilmaz et al., 2016).

#### 3.1.12 Silymarin

Silymarin, a lipophilic extract from the seeds of Silybum marianum, is protective against ototoxicity by inhibiting the expression of caspase-3 and PARP in cisplatin-induced cleavage through mechanisms such as scavenging of free radicals, reduction of ROS formation, and inhibition of fatty acid peroxidation (Cho et al., 2014b).

### 3.2 Non-flavonoids

#### 3.2.1 Ferulic acid (FA)

FA belongs to the phenolic acid family and is abundant in fruits and vegetables.FA acts as a potent antioxidant by up-regulating the cytoprotective system for scavenging free radicals and enhancing cellular stress response (Mancuso and Santangelo, 2014).FA inhibits cisplatin-induced cytotoxicity by blocking ROS formation and inducing endogenous antioxidant production, which in turn inhibits cisplatin-induced cytotoxicity (Jo et al., 2019). It exhibits antioxidant and otoprotective activity in the cochlea by up-regulating the Nrf-2/HO-1 pathway, while down-regulating p53 phosphorylation.Notably, this action of FA exhibits a biphasic response: it is pro-oxidant at lower concentrations and antioxidant at higher concentrations (Paciello et al., 2020). This unique concentration-dependent effect provides a richer direction for thinking about the application and research of ferulic acid in related fields.

#### 3.2.2 Resveratrol

Resveratrol belongs to the group of stilbenes and is widely found in a variety of plants such as berries, grapes and nuts. Animal experiments have confirmed the protective effect of resveratrol against cisplatin-induced ototoxicity (Yumusakhuylu et al., 2012; Simsek et al., 2019). With progressive research, it has been found that resveratrol significantly reduces cisplatin-induced increase in ROS (Lee S. K. et al., 2015), thus inhibiting cisplatin-induced cytotoxicity. The auriculoprotective effect of low-dose RV may be mediated by the anti-inflammatory effects of NFκB, IL6, and IL1β, as well as the antioxidant effects of CYP1A1 and the intracytoplasmic receptor for advanced glycation endproducts (RAGE).However, high doses of RV were not otoprotective in rats with cisplatin-induced hearing loss (Lee et al., 2020). In experiments targeting a mouse model of hearing loss and HEI-OC1 cells, resveratrol was found to upregulate miR-455-5p, which in turn downregulated phosphatase and tensin homolog (PTEN) and activated the phosphatidylinositol 3-kinase-protein kinase B (PI3K-Akt) signaling pathway in order to counteract cisplatin ototoxicity (Liu et al., 2021). Further studies demonstrated that growth arrest-specific transcript 5 (GAS5) could act as a molecular sponge for miR-455-5p and regulate miR-455-5p/PTEN expression to counteract cisplatin-induced apoptosis and ROS production (Wu et al., 2024).

#### 3.2.3 Schisandrin B (SchB)

SchB is one of the most prevalent and potent naturally occurring lignan monomers in Schisandrin B. It has a variety of pharmacological effects, including antioxidant, anti-inflammatory, and anti-apoptotic effects. In cisplatin-induced mouse experiments, treatment with SchB was able to enhance the survival of cochlear hair cells and effectively prevent apoptosis, as well as reduce cisplatin-induced ROS production (Li et al., 2024).

#### 3.2.4 Polyphenol

Curcumin is a phytochemical isolated from turmeric that has the ability to enhance the antitumour activity of cisplatin and reduce its side effects (Abadi et al., 2022), can directly scavenge free radicals and also enhance endogenous antioxidant enzyme activity by activating the Nrf2 pathway and inducing HO-1 expression to achieve antioxidant effects, which in turn reduce hearing damage (Fetoni et al., 2014).When curcumin is administered together with cisplatin, it exhibits different mechanisms of action depending on the cellular environment and dose, exerting dual antioxidant and pro-oxidant effects (Arwanda et al., 2023). Curcumin may enhance the anti-tumour effect of cisplatin by promoting a significant increase in the pro-apoptotic expression of Bax, inhibiting STAT3 phosphorylation, nuclear translocation of Nrf-2, and expression of NF-κB; and activating the Nrf-2/HO-1 signalling pathway, inhibiting p53 phosphorylation, expression of NF-κB, and enhancing endogenous antioxidant defence mechanisms to counteract cisplatin-induced ototoxicity (Paciello et al., 2020; Fetoni et al., 2015).The above studies have confirmed that curcumin has a significant dose-effect relationship, and there is an optimal concentration window--it can synergistically enhance the anticancer efficacy of cisplatin and minimize its toxic side effects.

Honokiol (HNK) is a multifunctional polyphenol derived from an Asian herb (Magnolia bark), HNK prevents cisplatin ototoxicity *in vitro* and *in vivo* and does not interfere with cisplatin treatment in homozygous mice (Tan et al., 2020). This is attributed to the direct activation of the protein sirtuin 3 (SIRT3) by HNK, which promotes ROS reduction and detoxification.

Ecklonia Cava Polyphenol (ECP) extract is a polyphenolic compound taken from brown algae living in the Far Eastern seas. It has a protective effect against cisplatin-induced cell death by reducing particularly reactive oxygen radicals and increasing the levels of enzymes such as catalase and glutathione peroxidase (Düzenli et al., 2016).

Caffeic acid, the active component of honey bee propolis extract, reduced intracellular ROS production through free radical scavenging activity; and decreased the expression of caspase-3, inhibited apoptosis, and ultimately attenuated cisplatin-induced hair cell loss in the HEI-OC1 cell line (Choi et al., 2014).

Pomegranate extract (PE) is rich in polyphenols and exhibits strong antioxidant activity (Sahin et al., 2025). Oral administration of PE has been shown to have a protective effect on the cochlea against cisplatin toxicity in rats (Yazici et al., 2012).

Anthocyanins is an extract of Vaccinium myrtillus, which presents antioxidant effects at high doses, thus reducing oxidative stress indices and preventing cell degeneration and protecting hearing from cisplatin-induced ototoxicity (Özdemir et al., 2019).

## 4 Discussion

The protective mechanism of polyphenols against cisplatin ototoxicity is mainly to build two major protective barriers in normal tissues. The first is the antioxidant barrier: polyphenols establish antioxidant defense by activating the Nrf2 signaling pathway, and Nrf2 upregulates the expression of HO-1, SOD-1, NQO-1 and other oxidative genes, so as to increase the number of antioxidant enzymes (SOD/CAT/GPX/GSH), and to neutralize cisplatin-induced reactive oxygen species (ROS) and reactive nitrogen species (RNS). Reactive ROS and RNS to reduce ROS accumulation, mitochondrial damage, inflammatory response and ferroptosis, and polyphenols also regulate the Nrf2 by activating the PI3K/AKT signaling pathway. The second is the inflammation-apoptosis dual inhibition barrier: blocking the NF-κB/p38-MAPK/COX-2/iNOS inflammatory cascade, attenuating the inflammatory response through the dual regulation of STAT3/STAT1 and inhibiting the JNK/p53-mediated pro-apoptotic signaling and regulating the balance of Bcl-2/Bax/caspase-3, which is the most effective way to reduce the inflammatory response in mitochondria and ferroptosis. (Zhang et al., 2025; Maiuolo et al., 2022; Li et al., 2023b; Stankovic et al., 2020; Gill et al., 2024).

Polyphenols face significant bioavailability bottlenecks when administered orally, and their pharmacokinetic properties make it difficult to reach therapeutic window concentrations in inner ear hair cells and auditory nerves (Osakabe et al., 2023), a limitation that significantly restricts their potential application in the prevention and treatment of CIO.However, reactive oxygen species present a dual role in the pathological process of CIO: as a key pathogenic factor mediating cisplatin ototoxicity and as a pharmacological basis for its tumor cell-killing effects, and this paradoxical property makes the construction of an inner-ear-targeted delivery system a central challenge for synergistic chemotherapy with polyphenolic compounds.

Recent breakthroughs in nano-delivery systems have provided innovative strategies to address this challenge (Thakur et al., 2024). Nano-embedded curcumin not only significantly improves cell survival and attenuates the ototoxicity of cisplatin through enhanced bioavailability and targeted delivery (Salehi et al., 2014), but also synergistically enhances the anticancer efficacy of cisplatin in combination with cisplatin while improving curcumin bioavailability (Sandhiutami et al., 2021). In addition resveratrol has low bioavailability due to its poor water solubility, low stability and rapid metabolism. Some studies have demonstrated that different types of resveratrol nanoparticles can significantly improve its therapeutic potential in a variety of diseases such as neurodegenerative diseases, cancer, diabetes, etc. (Chung et al., 2020).EGCG is a promising natural compound that enhances the efficacy of chemotherapy and reduces side effects through multiple pathways, and combining it with nanotechnology can break through the limitations of poor bioavailability and targeting to significantly enhance the therapeutic effect (Wang X. et al., 2023). Tetrahedral DNA nanostructures (TDNs) as carriers loaded with antioxidant EGCG can efficiently penetrate the biological barrier, sustained release of drugs and antioxidant effects, and significantly improve the noise-induced hearing loss, which provides a novel delivery system for the treatment of inner ear diseases (Chen et al., 2022).

Different types of polyphenols may have different mechanisms in preventing cisplatin ototoxicity, and may act synergistically in enhancing its anticancer activity to reduce side effects.EGCG has antioxidant, anti-inflammatory, and inhibitory effects on Caspase-1 and NF-κB, and curcumin regulates the Bcl-2/BAX ratio, inhibits the mitochondrial apoptotic pathway, and reduces Caspase-3 activation. The superimposition of these two effects enhances the anti-apoptotic effect and reduces the dosage requirement of a single component, providing a potential solution for adjuvant therapy to cisplatin chemotherapy, reducing side effects and improving the quality of life of patients (Primadewi et al., 2023). Potential interactions between polyphenols and common drugs used in cisplatin combination chemotherapy also need to be noted. One study demonstrated that curcumin combined with paclitaxel treatment was superior to paclitaxel-placebo combination therapy in terms of ORR and physical performance after 12 weeks of treatment, and there were also no major safety concerns or reduction in quality of life (Saghatelyan et al., 2020).EGCG synergistically enhances the efficacy of drugs such as 5 - Fluorouracil and cisplatin, and reduces cardiac, renal, and other organ EGCG may synergistically enhance the efficacy of drugs such as 5-fluorouracil and cisplatin and reduce the toxicity in the heart and kidney, but some drugs (e.g., bortezomib, sunitinib) may antagonize EGCG, but clinical evidence is limited, and further validation of their safety and efficacy is needed (Lecumberri et al., 2013).

Despite their low bioavailability, oral polyphenols can modulate the intestinal flora through their antimicrobial properties, increasing the proportion of beneficial bacteria in the gut and reducing pathogenic bacteria (Scazzocchio et al., 2020). Improvement in the balance of intestinal flora helps to maintain the integrity of the intestinal barrier, reduces the influx of harmful substances into the body, and inhibits inflammatory responses (Liu et al., 2024). It has been reported that polyphenols ameliorate certain diseases by modulating the intestinal flora (Da C Pinaffi-Langley et al., 2024; Sarubbo et al., 2023). At the same time, we have come to recognize the association between the gut-inner ear axis and hearing loss (Guo et al., 2024; Yin et al., 2023). Based on this, the topic of whether orally administered polyphenols can ameliorate hearing loss by modulating the intestinal flora is of significant research value.

## 5 Conclusion

Cisplatin is a highly effective and broad-spectrum anticancer drug, but its severe ototoxicity limits its clinical application, and its mechanism involves various pathways such as DNA damage, oxidative stress, inflammatory response, mitochondrial dysfunction, and ferroptosis. Polyphenols show promise in mitigating cisplatin-induced damage to Corti organs, spiral ganglion neurons, and vascular striatum by scavenging free radicals, inhibiting inflammatory factors, and regulating apoptosis-related proteins. However, several challenges remain, including low bioavailability, an incomplete understanding of their mechanisms, uncertainties about the optimal dosing regimen, and the potential interactions among different polyphenols. Further studies should explore the optimal strategy for the combined application of polyphenols and cisplatin to provide new ideas and methods for clinical prevention and treatment of cisplatin ototoxicity.

### 5.1 Future perspectives

Cisplatin, as a commonly used chemotherapeutic agent, is highly effective, but the problem of CIO seriously affects patients’ quality of life. Polyphenols have shown impressive dual potentials in preventing CIO and enhancing the efficacy of chemotherapy, and are expected to be a key factor in improving the status of cisplatin chemotherapy. However, polyphenols still face many obstacles in moving from basic research to clinical application.

Currently, most of the relevant studies remain in the preclinical stage, and the lack of key pharmacokinetic data for clinical application, such as effective dose, bioavailability and other information, restricts the development of rational drug regimens. In terms of drug delivery strategies, the emergence of nano drug delivery systems holds promise for improving the oral utilization of polyphenols. However, this system still has large optimization aspects in terms of shape, size, and targeted delivery efficiency, which is still far from the ideal state of precision and efficiency. In addition, many important issues need to be further explored, such as the mechanism of the superimposed effect and its safety when polyphenols are applied in combination, the possible interactions with other chemotherapeutic agents such as 5 - fluorouracil, paclitaxel analogs, gemcitabine, pemetrexed, etc., as well as whether oral polyphenols have an impact on ototoxicity by regulating the intestinal flora.

Addressing the above issues has immeasurable clinical value in improving the comprehensive efficacy of cisplatin chemotherapy and effectively reducing the risk of ototoxicity. It will not only bring better treatment experience for patients, but also promote new breakthroughs in the field of anti-cancer therapy.

## References

[B1] Abadi A. J. Mirzaei S. Mahabady M. K. Hashemi F. Zabolian A. Hashemi F. (2022). Curcumin and its derivatives in cancer therapy: potentiating antitumor activity of cisplatin and reducing side effects. Phytotherapy Res. PTR 36 (1), 189–213. 10.1002/ptr.7305 34697839

[B2] Abotaleb M. Liskova A. Kubatka P. Büsselberg D. (2020). Therapeutic potential of plant phenolic acids in the treatment of cancer. Biomolecules 10 (2), 221. 10.3390/biom10020221 32028623 PMC7072661

[B3] Anantharaju P. G. Gowda P. C. Vimalambike M. G. Madhunapantula S. V. (2016). An overview on the role of dietary phenolics for the treatment of cancers. Nutr. J. 15 (1), 99. 10.1186/s12937-016-0217-2 27903278 PMC5131407

[B4] Anfuso C. D. Cosentino A. Agafonova A. Zappalà A. Giurdanella G. Trovato Salinaro A. (2022). Pericytes of stria Vascularis are targets of cisplatin-induced ototoxicity: new insights into the molecular mechanisms involved in blood-labyrinth barrier breakdown. Int. J. Mol. Sci. 23 (24), 15790. 10.3390/ijms232415790 36555432 PMC9781621

[B5] Arwanda M. D. Haryuna T. S. Adriztina I. Khalid K. (2023). Curcumin prevents ototoxicity induced by cisplatin as evaluated with OAE. Iran. J. otorhinolaryngology 35 (129), 189–197. 10.22038/IJORL.2023.71786.3452 PMC10368170 37497164

[B6] Babolmorad G. Latif A. Domingo I. K. Pollock N. M. Delyea C. Rieger A. M. (2021). Toll-like receptor 4 is activated by platinum and contributes to cisplatin-induced ototoxicity. EMBO Rep. 22 (5), e51280. 10.15252/embr.202051-280 33733573 PMC8097357

[B7] Başoğlu M. S. Eren E. Aslan H. Bingölballı A. G. Oztürkcan S. Katılmış H. (2012). Prevention of cisplatin ototoxicity: efficacy of micronized flavonoid fraction in a Guinea pig model. Int. J. Pediatr. otorhinolaryngology 76 (9), 1343–1346. 10.1016/j.ijporl.2012.06.003 22763210

[B8] Benkafadar N. Menardo J. Bourien J. Nouvian R. François F. Decaudin D. (2017). Reversible p53 inhibition prevents cisplatin ototoxicity without blocking chemotherapeutic efficacy. EMBO Mol. Med. 9 (1), 7–26. 10.15252/emmm.201606230 27794029 PMC5210089

[B9] Bhatta P. Dhukhwa A. Sheehan K. Al Aameri R. F. H. Borse V. Ghosh S. (2019). Capsaicin protects against cisplatin ototoxicity by changing the STAT3/STAT1 ratio and activating cannabinoid (CB2) receptors in the cochlea. Sci. Rep. 9 (1), 4131. 10.1038/s41598-019-40425-9 30858408 PMC6411993

[B10] Borse V. Al Aameri R. F. H. Sheehan K. Sheth S. Kaur T. Mukherjea D. (2017). Epigallocatechin-3-gallate, a prototypic chemopreventative agent for protection against cisplatin-based ototoxicity. Cell death and Dis. 8 (7), e2921. 10.1038/cddis.2017.314 PMC5550861 28703809

[B11] Bouyahya A. Omari N. E. Bakrim S. Hachlafi N. E. Balahbib A. Wilairatana P. (2022). Advances in dietary phenolic compounds to improve chemosensitivity of anticancer drugs. Cancers 14 (19), 4573. 10.3390/cancers14194573 36230494 PMC9558505

[B12] Callejo A. Sedó-Cabezón L. Juan I. D. Llorens J. (2015). Cisplatin-induced ototoxicity: effects, mechanisms and protection strategies. Toxics 3 (3), 268–293. 10.3390/toxics3030268 29051464 PMC5606684

[B13] Cederroth C. R. Dyhrfjeld-Johnsen J. Canlon B. (2024). Pharmacological approaches to hearing loss. Pharmacol. Rev. 76 (6), 1063–1088. 10.1124/pharmrev.124.001195 39164117 PMC11549935

[B14] Chen Y. Gu J. Liu Y. Xu K. Song J. Wang X. (2022). Epigallocatechin gallate-loaded tetrahedral DNA nanostructures as a novel inner ear drug delivery system. Nanoscale 14 (22), 8000–8011. 10.1039/d1nr07921b 35587814

[B15] Cheynier V. (2005). Polyphenols in foods are more complex than often thought. Am. J. Clin. Nutr. 81 (1 Suppl. l), 223S–229S. 10.1093/ajcn/81.1.223S 15640485

[B16] Cho S. I. Lee J. E. Do N. Y. (2014a). Protective effect of silymarin against cisplatin-induced ototoxicity. Int. J. Pediatr. otorhinolaryngology 78 (3), 474–478. 10.1016/j.ijporl.2013.12.024 24434130

[B17] Cho S. I. Lee J. H. Park J. H. Do N. Y. (2014b). Protective effect of (-)-epigallocatechin-3-gallate against cisplatin-induced ototoxicity. J. laryngology otology 128 (4), 350–355. 10.1017/S0022215114000553 24735939

[B18] Choi B. M. Chen X. Y. Gao S. S. Zhu R. Kim B. R. (2011). Anti-apoptotic effect of phloretin on cisplatin-induced apoptosis in HEI-OC1 auditory cells. Pharmacol. Rep. 63, 708–716. 10.1016/s1734-1140(11)70582-5 21857081

[B19] Choi J. Kim S. H. Rah Y. C. Chae S. W. Lee J. D. Lee B. D. (2014). Effects of caffeic acid on cisplatin-induced hair cell damage in HEI-OC1 auditory cells. Int. J. Pediatr. otorhinolaryngology 78 (12), 2198–2204. 10.1016/j.ijporl.2014.10.013 25458160

[B20] Chung I.-M. Subramanian U. Thirupathi P. Venkidasamy B. Samynathan R. Gangadhar B. H. (2020). Resveratrol nanoparticles: a promising therapeutic advancement over native resveratrol. Processes 8 (4), 458. 10.3390/pr8040458

[B21] Crona D. J. Faso A. Nishijima T. F. McGraw K. A. Galsky M. D. Milowsky M. I. (2017). A systematic review of strategies to prevent cisplatin-induced nephrotoxicity. Oncol. 22 (5), 609–619. 10.1634/theoncologist.2016-0319 PMC5423518 28438887

[B22] Da C Pinaffi-Langley A. C. Tarantini S. Hord N. G. Yabluchanskiy A. (2024). Polyphenol-derived microbiota metabolites and cardiovascular health: a concise review of human studies. Antioxidants Basel, Switz. 13 (12), 1552. 10.3390/antiox13121552 PMC11673714 39765880

[B23] Dai D. Chen C. Lu C. Guo Y. Li Q. Sun C. (2024). Apoptosis, autophagy, ferroptosis, and pyroptosis in cisplatin-induced ototoxicity and protective agents. Front. Pharmacol. 15, 1430469. 10.3389/fphar.2024.1430469 39380912 PMC11459463

[B24] Dalgaard F. Bondonno N. P. Murray K. Bondonno C. P. Lewis J. R. Croft K. D. (2019). Associations between habitual flavonoid intake and hospital admissions for atherosclerotic cardiovascular disease: a prospective cohort study. Lancet. Planet. health 3 (11), e450–e459. 10.1016/S2542-5196(19)30212-8 31777336

[B25] Dias M. A. Sampaio A. L. Venosa A. R. Meneses E. deA. Oliveira C. A. (2015). The chemopreventive effect of Ginkgo biloba extract 761 against cisplatin ototoxicity: a pilot study. Int. tinnitus J. 19 (2), 12–19. 10.5935/0946-5448.20150003 27186927

[B26] Düzenli U. Olgun Y. Aktaş S. Pamukoğlu A. Altun Z. (2016). Effect of ecklonia Cava polyphenol extract in house ear institute-organ of Corti 1 cells against cisplatin ototoxicity: a preliminary study. Turkish archives otorhinolaryngology 54 (4), 141–145. 10.5152/tao.2016.1974 PMC5782951 29392035

[B27] Elmorsy E. A. Saber S. Hamad R. S. Abdel-Reheim M. A. El-Kott A. F. AlShehri M. A. (2024). Advances in understanding cisplatin-induced toxicity: molecular mechanisms and protective strategies. Eur. Fed. Pharm. Sci. 203, 106939. 10.1016/j.ejps.2024.106939 39423903

[B28] Eryilmaz A. Eliyatkin N. Demirci B. Basal Y. Kurt Omurlu I. Gunel C. (2016). Protective effect of Pycnogenol on cisplatin-induced ototoxicity in rats. Pharm. Biol. 54 (11), 2777–2781. 10.1080/13880209.2016.1177093 27158843

[B29] Fetoni A. R. Eramo S. L. Paciello F. Rolesi R. Podda M. V. Troiani D. (2014). Curcuma longa (curcumin) decreases *in vivo* cisplatin-induced ototoxicity through heme oxygenase-1 induction. Otology and Neurotol. 35 (5), e169–e177. 10.1097/MAO.0000000000000302 24608370

[B30] Fetoni A. R. Paciello F. Mezzogori D. Rolesi R. Eramo S. L. Paludetti G. (2015). Molecular targets for anticancer redox chemotherapy and cisplatin-induced ototoxicity: the role of curcumin on pSTAT3 and Nrf-2 signalling. Br. J. cancer 113 (10), 1434–1444. 10.1038/bjc.2015.359 26469832 PMC4815880

[B31] Fetoni A. R. Paciello F. Troiani D. (2022). Cisplatin chemotherapy and cochlear damage: otoprotective and chemosensitization properties of polyphenols. Antioxidants and redox Signal. 36 (16-18), 1229–1245. 10.1089/ars.2021.0183 34731023

[B32] Gill N. B. Dowker-Key P. D. Hedrick M. Bettaieb A. (2024). Unveiling the role of oxidative stress in cochlear hair cell death: prospective phytochemical therapeutics against sensorineural hearing loss. Int. J. Mol. Sci. 25 (8), 4272. 10.3390/ijms25084272 38673858 PMC11050722

[B33] Gong T. Liu L. Jiang W. Zhou R. (2020). DAMP-sensing receptors in sterile inflammation and inflammatory diseases. Nat. Rev. Immunol. 20 (2), 95–112. 10.1038/s41577-019-0215-7 31558839

[B34] Grabska-Kobyłecka I. Szpakowski P. Król A. Książek-Winiarek D. Kobyłecki A. Głąbiński A. (2023). Polyphenols and their impact on the prevention of neurodegenerative diseases and development. Nutrients 15 (15), 3454. 10.3390/nu15153454 37571391 PMC10420887

[B35] Gündoğdu R. Erkan M. Aydın M. Sönmez M. F. Vural A. Kökoğlu K. (2019). Assessment of the effectiveness of quercetin on cisplatin-induced ototoxicity in rats. J. Int. Adv. otology 15 (2), 229–236. 10.5152/iao.2019.5902 PMC6750792 31287434

[B36] Guo Z. Wu Y. Chen B. Kong M. Xie P. Li Y. (2024). Superparamagnetic iron oxide nanoparticle regulates microbiota-gut-inner ear axis for hearing protection. Natl. Sci. Rev. 11 (6), nwae100. 10.1093/nsr/nwae100 38707203 PMC11067960

[B37] Hong S. J. Dawson T. M. Dawson V. L. (2004). Nuclear and mitochondrial conversations in cell death: PARP-1 and AIF signaling. Trends Pharmacol. Sci. 25 (5), 259–264. 10.1016/j.tips.2004.03.005 15120492

[B38] Honkura Y. Matsuo H. Murakami S. Sakiyama M. Mizutari K. Shiotani A. (2016). NRF2 is a key target for prevention of noise-induced hearing loss by reducing oxidative damage of cochlea. Sci. Rep. 6, 19329. 10.1038/srep19329 26776972 PMC4726010

[B39] Huang T. L. Jiang W. J. Zhou Z. Shi T. F. Yu M. Yu M. (2024). Quercetin attenuates cisplatin-induced mitochondrial apoptosis via PI3K/Akt mediated inhibition of oxidative stress in pericytes and improves the blood labyrinth barrier permeability. Chemico-biological Interact. 393, 110939. 10.1016/j.cbi.2024.110939 38490643

[B40] Jakobušić Brala C. Karković Marković A. Kugić A. Torić J. Barbarić M. (2023). Combination chemotherapy with selected polyphenols in preclinical and clinical studies-an update overview. Mol. Basel, Switz. 28 (9), 3746. 10.3390/molecules28093746 PMC10180288 37175156

[B41] Jo E. R. Youn C. K. Jun Y. Cho S. I. (2019). The protective role of ferulic acid against cisplatin-induced ototoxicity. Int. J. Pediatr. otorhinolaryngology 120, 30–35. 10.1016/j.ijporl.2019.02.001 30753979

[B42] Johnstone T. C. Suntharalingam K. Lippard S. J. (2016). The next generation of platinum drugs: targeted Pt(II) agents, nanoparticle delivery, and Pt(IV) prodrugs. Chem. Rev. 116 (5), 3436–3486. 10.1021/acs.chemrev.5b00597 26865551 PMC4792284

[B43] Kara M. Türkön H. Karaca T. Güçlü O. Uysal S. Türkyılmaz M. (2016). Evaluation of the protective effects of hesperetin against cisplatin-induced ototoxicity in a rat animal model. Int. J. Pediatr. otorhinolaryngology 85, 12–18. 10.1016/j.ijporl.2016.03.019 27240489

[B44] Kaur T. Mukherjea D. Sheehan K. Jajoo S. Rybak L. P. Ramkumar V. (2011). Short interfering RNA against STAT1 attenuates cisplatin-induced ototoxicity in the rat by suppressing inflammation. Cell death and Dis. 2 (7), e180. 10.1038/cddis.2011.63 PMC3199718 21776018

[B45] Kim C. H. Kang S. U. Pyun J. Lee M. H. Hwang H. S. Lee H. (2008). Epicatechin protects auditory cells against cisplatin-induced death. Apoptosis Int. J. Program. cell death 13 (9), 1184–1194. 10.1007/s10495-008-0242-5 18670884

[B46] Kim S. J. Lee J. H. Kim B. S. So H. S. Park R. Myung N. Y. (2012). (-)-Epigallocatechin-3-gallate protects against NO-induced ototoxicity through the regulation of caspase- 1, caspase-3, and NF-κB activation. PloS one 7 (9), e43967. 10.1371/journal.pone.0043967 23028481 PMC3461011

[B47] Ko J. H. Sethi G. Um J. Y. Shanmugam M. K. Arfuso F. Kumar A. P. (2017). The role of resveratrol in cancer therapy. Int. J. Mol. Sci. 18 (12), 2589. 10.3390/ijms18122589 29194365 PMC5751192

[B48] Kong S. Xiao Y. Chen L. Jin Y. Qiao R. Xu K. (2024). Apigenin attenuates cisplatin-induced hair cell damage in the zebrafish lateral line. Food Chem. Toxicol. 194, 115099. 10.1016/j.fct.2024.115099 39521239

[B49] Kucharava K. Sekulic-Jablanovic M. Horvath L. Bodmer D. Petkovic V. (2019). Pasireotide protects mammalian cochlear hair cells from gentamicin ototoxicity by activating the PI3K-Akt pathway. Cell death and Dis. 10 (2), 110. 10.1038/s41419-019-1386-7 PMC6365508 30728348

[B50] Lecumberri E. Dupertuis Y. M. Miralbell R. Pichard C. (2013). Green tea polyphenol epigallocatechin-3-gallate (EGCG) as adjuvant in cancer therapy. Clin. Nutr. Edinb. Scotl. 32 (6), 894–903. 10.1016/j.clnu.2013.03.008 23582951

[B51] Lee C. H. Kim K. W. Lee S. M. Kim S. Y. (2020). Dose-dependent effects of resveratrol on cisplatin-induced hearing loss. Int. J. Mol. Sci. 22 (1), 113. 10.3390/ijms22010113 33374326 PMC7794979

[B52] Lee J. S. Kang S. U. Hwang H. S. Pyun J. H. Choung Y. H. Kim C. H. (2010). Epicatechin protects the auditory organ by attenuating cisplatin-induced ototoxicity through inhibition of ERK. Toxicol. Lett. 199 (3), 308–316. 10.1016/j.toxlet.2010.09.013 20883750

[B53] Lee S. H. Kim H. S. An Y. S. Chang J. Choi J. Im G. J. (2015a). Protective effect of resveratrol against cisplatin-induced ototoxicity in HEI-OC1 auditory cells. Int. J. Pediatr. otorhinolaryngology 79 (1), 58–62. 10.1016/j.ijporl.2014.11.008 25434479

[B54] Lee S. K. Oh K. H. Chung A. Y. Park H. C. Lee S. H. Kwon S. Y. (2015b). Protective role of quercetin against cisplatin-induced hair cell damage in zebrafish embryos. Hum. and Exp. Toxicol. 34 (11), 1043–1052. 10.1177/0960327114567766 25591968

[B55] Li W. Guo Y. Zhang C. Wu R. Yang A. Y. Gaspar J. (2016). Dietary phytochemicals and cancer chemoprevention: a perspective on oxidative stress, inflammation, and epigenetics. Chem. Res. Toxicol. 29 (12), 2071–2095. 10.1021/acs.chemrestox.6b00413 27989132

[B56] Li Y. Liu Z. Chen J. Wang R. An X. Tian C. (2024). Schisandrin B protect inner hair cells from cisplatin by inhibiting celluar oxidative stress and apoptosis. Toxicol. vitro Int. J. Publ. Assoc. BIBRA 99, 105852. 10.1016/j.tiv.2024.105852 38789064

[B57] Li Y. Wu J. Yu H. Lu X. Ni Y. (2023b). Formononetin ameliorates cisplatin-induced hair cell death via activation of the PI3K/AKT-Nrf2 signaling pathway. Heliyon 10 (1), e23750. 10.1016/j.heliyon.2023.e23750 38192850 PMC10772176

[B58] Li Y. Zhang T. Song Q. Gao D. Li Y. Jie H. (2023a). Cisplatin ototoxicity mechanism and antagonistic intervention strategy: a scope review. Front. Cell. Neurosci. 17, 1197051. 10.3389/fncel.2023.1197051 37323582 PMC10267334

[B59] Liao Y. Mao H. Gao X. Lin H. Li W. Chen Y. (2024). Drug screening identifies aldose reductase as a novel target for treating cisplatin-induced hearing loss. Free Radic. Biol. and Med. 210, 430–447. 10.1016/j.freeradbiomed.2023.11.025 38056576

[B60] Lin Y. Liang R. Xie K. Ma T. Zhang J. Xu T. (2024). Puerarin inhibits cisplatin-induced ototoxicity in mice through regulation of TRPV1-dependent calcium overload. Biochem. Pharmacol. 220, 115962. 10.1016/j.bcp.2023.115962 38043717

[B61] Liu Y. Fang M. Tu X. Mo X. Zhang L. Yang B. (2024). Dietary polyphenols as anti-aging agents: targeting the hallmarks of aging. Nutrients 16 (19), 3305. 10.3390/nu16193305 39408272 PMC11478989

[B62] Liu Y. Wu H. Zhang F. Yang J. He J. (2021). Resveratrol upregulates miR-455-5p to antagonize cisplatin ototoxicity via modulating the PTEN-PI3K-AKT axis. Biochem. cell Biol. = Biochimie Biol. Cell. 99 (3), 385–395. 10.1139/bcb-2020-0459 34077275

[B63] Lou J. Wu F. He W. Hu R. Cai Z. Chen G. (2024). Hesperidin activates Nrf2 to protect cochlear hair cells from cisplatin-induced damage. Redox Rep. Commun. free Radic. Res. 29 (1), 2341470. 10.1080/13510002.2024.2341470 PMC11025410 38629504

[B64] Lu X. Deng T. Dong H. Han J. Yu Y. Xiang D. (2022). Novel application of eupatilin for effectively attenuating cisplatin-induced auditory hair cell death via mitochondrial apoptosis pathway. Oxidative Med. Cell. Longev. 2022, 1090034. 10.1155/2022/1090034 PMC8786471 35082962

[B65] Lu X. Yin N. Chen C. Zhou Y. Ji L. Zhang B. (2024). Mangiferin alleviates cisplatin-induced ototoxicity in sensorineural hearing loss. Biomed. and Pharmacother. = Biomedecine and Pharmacother. 178, 117174. 10.1016/j.biopha.2024.117174 39098177

[B66] Lv X. Yang C. Li X. Liu Y. Yang Y. Jin T. (2025). Ferroptosis and hearing loss: from molecular mechanisms to therapeutic interventions. J. enzyme inhibition Med. Chem. 40 (1), 2468853. 10.1080/14756366.2025.2468853 PMC11852237 39992186

[B67] Ma Q. (2013). Role of nrf2 in oxidative stress and toxicity. Annu. Rev. Pharmacol. Toxicol. 53, 401–426. 10.1146/annurev-pharmtox-011112-140320 23294312 PMC4680839

[B68] Maiuolo J. Musolino V. Gliozzi M. Carresi C. Oppedisano F. Nucera S. (2022). The employment of genera *Vaccinium, citrus, olea,* and *Cynara*Polyphenols for the reduction of selected anti-cancer drug side effects. Nutrients 14 (8), 1574. 10.3390/nu14081574 35458136 PMC9025632

[B69] Mancuso C. Santangelo R. (2014). Ferulic acid: pharmacological and toxicological aspects. Food Chem. Toxicol. 65, 185–195. 10.1016/j.fct.2013.12.024 24373826

[B70] Mukherjea D. Jajoo S. Sheehan K. Kaur T. Sheth S. Bunch J. (2011). NOX3 NADPH oxidase couples transient receptor potential vanilloid 1 to signal transducer and activator of transcription 1-mediated inflammation and hearing loss. Antioxidants and redox Signal. 14 (6), 999–1010. 10.1089/ars.2010.3497 PMC3043978 20712533

[B71] Osakabe N. Modafferi S. Ontario M. L. Rampulla F. Zimbone V. Migliore M. R. (2023). Polyphenols in inner ear neurobiology, health and disease: from bench to clinics. Med. Kaunas. Lith. 59 (11), 2045. 10.3390/medicina59112045 PMC10673256 38004094

[B72] Özdemir D. Özgür A. Kalkan Y. Terzi S. Tümkaya L. Yılmaz A. (2019). The protective effects of whortleberry extract against cisplatin-induced ototoxicity in rats. Braz. J. otorhinolaryngology 85 (1), 55–62. 10.1016/j.bjorl.2017.10.009 PMC9442816 29174583

[B73] Paciello F. Fetoni A. R. Mezzogori D. Rolesi R. Di Pino A. Paludetti G. (2020). The dual role of curcumin and ferulic acid in counteracting chemoresistance and cisplatin-induced ototoxicity. Sci. Rep. 10 (1), 1063. 10.1038/s41598-020-57965-0 31974389 PMC6978317

[B75] Primadewi N. Kariosentono H. Probandari A. Wiboworini B. (2023). The effect of combination between green tea extract and curcumin extract from Mt. Lawu on BAX, bcl-2 and caspase-3 in cisplatin-induced rat models. Pharmacogn. J. 15 (2), 370–374. 10.5530/pj.2023.15.57

[B76] Ramkumar V. Mukherjea D. Dhukhwa A. Rybak L. P. (2021). Oxidative stress and inflammation caused by cisplatin ototoxicity. Antioxidants Basel, Switz. 10 (12), 1919. 10.3390/antiox10121919 PMC8750101 34943021

[B77] Rose O. Croonenberg T. Clemens S. Hinteregger T. Eppacher S. Huber-Cantonati P. (2024). Cisplatin-induced hearing loss, oxidative stress, and antioxidants as a therapeutic strategy-A state-of-the-art review. Antioxidants Basel, Switz. 13 (12), 1578. 10.3390/antiox13121578 PMC11673797 39765905

[B78] Rybak L. P. Mukherjea D. Ramkumar V. (2019). Mechanisms of cisplatin-induced ototoxicity and prevention. Seminars Hear. 40 (2), 197–204. 10.1055/s-0039-1684048 PMC6486366 31036996

[B79] Saghatelyan T. Tananyan A. Janoyan N. Tadevosyan A. Petrosyan H. Hovhannisyan A. (2020). Efficacy and safety of curcumin in combination with paclitaxel in patients with advanced, metastatic breast cancer: a comparative, randomized, double-blind, placebo-controlled clinical trial. Phytomedicine Int. J. phytotherapy Phytopharm. 70, 153218. 10.1016/j.phymed.2020.153218 32335356

[B80] Sahin K. Sahin Aktura S. Bahceci I. Mercantepe T. Tumkaya L. Topcu A. (2025). Is *Punica granatum* efficient against sepsis? A comparative study of amifostine versus pomegranate. Life Basel, Switz. 15 (1), 78. 10.3390/life15010078 PMC11766669 39860018

[B81] Salehi P. Akinpelu O. V. Waissbluth S. Peleva E. Meehan B. Rak J. (2014). Attenuation of cisplatin ototoxicity by otoprotective effects of nanoencapsulated curcumin and dexamethasone in a Guinea pig model. Otological Soc. Am. Neurotol. 35 (7), 1131–1139. 10.1097/MAO.0000000000000403 24841915

[B82] Sandhiutami N. M. D. Arozal W. Louisa M. Rahmat D. Wuyung P. E. (2021). Curcumin nanoparticle enhances the anticancer effect of cisplatin by inhibiting PI3K/AKT and JAK/STAT3 pathway in rat ovarian carcinoma induced by DMBA. Front. Pharmacol. 11, 603235. 10.3389/fphar.2020.603235 33536913 PMC7848208

[B83] Santucci R. Sinibaldi F. Cozza P. Polticelli F. Fiorucci L. (2019). Cytochrome c: an extreme multifunctional protein with a key role in cell fate. Int. J. Biol. Macromol. 136, 1237–1246. 10.1016/j.ijbiomac.2019.06.180 31252007

[B84] Sarubbo F. Moranta D. Tejada S. Jiménez M. Esteban S. (2023). Impact of gut microbiota in brain ageing: polyphenols as beneficial modulators. Antioxidants Basel, Switz. 12 (4), 812. 10.3390/antiox12040812 PMC10134998 37107187

[B85] Scazzocchio B. Minghetti L. D'Archivio M. (2020). Interaction between gut microbiota and curcumin: a new key of understanding for the health effects of curcumin. Nutrients 12 (9), 2499. 10.3390/nu12092499 32824993 PMC7551052

[B86] Schmitt N. C. Rubel E. W. Nathanson N. M. (2009). Cisplatin-induced hair cell death requires STAT1 and is attenuated by epigallocatechin gallate. J. Neurosci. official J. Soc. Neurosci. 29 (12), 3843–3851. 10.1523/JNEUROSCI.5842-08.2009 PMC2707781 19321781

[B87] Si X. Yu Z. Ren X. Huang L. Feng Y. (2022). Efficacy and safety of standardized Ginkgo biloba L. leaves extract as an adjuvant therapy for sudden sensorineural hearing loss: a systematic review and meta-analysis. J. Ethnopharmacol. 282, 114587. 10.1016/j.jep.2021.114587 34474140

[B88] Simsek G. Taş B. M. Muluk N. B. Azman M. Kılıç R. (2019). Comparison of the protective efficacy between intratympanic dexamethasone and resveratrol treatments against cisplatin-induced ototoxicity: an experimental study. Eur. Fed. Oto-Rhino-Laryngological 276 (12), 3287–3293. 10.1007/s00405-019-05635-x 31531774

[B89] Song W. Zhang L. Cui X. Wang R. Ma J. Xu Y. (2024). Nobiletin alleviates cisplatin-induced ototoxicity via activating autophagy and inhibiting NRF2/GPX4-mediated ferroptosis. Sci. Rep. 14 (1), 7889. 10.1038/s41598-024-55614-4 38570541 PMC10991266

[B90] Stankovic J. S. K. Selakovic D. Mihailovic V. Rosic G. (2020). Antioxidant supplementation in the treatment of neurotoxicity induced by platinum-based chemotherapeutics—a review. Int. J. Mol. Sci. 21 (20), 7753. 10.3390/ijms21207753 33092125 PMC7589133

[B91] Stockwell B. R. Jiang X. Gu W. (2020). Emerging mechanisms and disease relevance of ferroptosis. Trends cell Biol. 30 (6), 478–490. 10.1016/j.tcb.2020.02.009 32413317 PMC7230071

[B92] Sung C. Y. W. Hayase N. Yuen P. S. T. Lee J. Fernandez K. Hu X. (2024). Macrophage depletion protects against cisplatin-induced ototoxicity and nephrotoxicity. Sci. Adv. 10 (30), eadk9878. 10.1126/sciadv.adk9878 39047106 PMC11268410

[B93] Tajdini A. Karimi Yazdi A. Ravand H. Sahebi L. (2023). The use of herbal medicine in sudden sensorineural hearing loss in diabetic patients. Iran. J. otorhinolaryngology 35 (129), 207–215. 10.22038/IJORL.2023.69813.3368 PMC10368171 37497158

[B94] Tan M. Toplu Y. Varan E. Sapmaz E. Özhan O. Parlakpınar H. (2022). The effect of genistein on cisplatin induced ototoxicity and oxidative stress. Braz. J. otorhinolaryngology 88 (1), 105–111. 10.1016/j.bjorl.2021.07.001 PMC9422515 34602350

[B95] Tan W. J. T. Song L. (2023). Role of mitochondrial dysfunction and oxidative stress in sensorineural hearing loss. Hear. Res. 434, 108783. 10.1016/j.heares.2023.108783 37167889

[B96] Tan W. J. T. Vlajkovic S. M. (2023). Molecular characteristics of cisplatin-induced ototoxicity and therapeutic interventions. Int. J. Mol. Sci. 24 (22), 16545. 10.3390/ijms242216545 38003734 PMC10671929

[B97] Tan X. Zhou Y. Agarwal A. Lim M. Xu Y. Zhu Y. (2020). Systemic application of honokiol prevents cisplatin ototoxicity without compromising its antitumor effect. Am. J. cancer Res. 10 (12), 4416–4434. 33415008 PMC7783741

[B98] Tang D. Chen X. Kang R. Kroemer G. (2021). Ferroptosis: molecular mechanisms and health implications. Cell Res. 31 (2), 107–125. 10.1038/s41422-020-00441-1 33268902 PMC8026611

[B99] Tang D. Wang X. Wu J. Li Y. Li C. Qiao X. (2024). Cinchonine and cinchonidine alleviate cisplatin-induced ototoxicity by regulating PI3K-AKT signaling. CNS Neurosci. and Ther. 30 (2), e14403. 10.1111/cns.14403 37577804 PMC10848099

[B100] Thakur N. S. Rus I. Herbert A. Zallocchi M. Chakrabarty B. Joshi A. D. (2024). Crosslinked-hybrid nanoparticle embedded in thermogel for sustained co-delivery to inner ear. J. nanobiotechnology 22 (1), 482. 10.1186/s12951-024-02686-z 39135039 PMC11321169

[B101] Un H. Ugan R. A. Gurbuz M. A. Bayir Y. Kahramanlar A. Kaya G. (2021). Phloretin and phloridzin guard against cisplatin-induced nephrotoxicity in mice through inhibiting oxidative stress and inflammation. Life Sci. 266, 118869. 10.1016/j.lfs.2020.118869 33309722

[B102] Vladu A. F. Ficai D. Ene A. G. Ficai A. (2022). Combination therapy using polyphenols: an efficient way to improve antitumoral activity and reduce resistance. Int. J. Mol. Sci. 23 (18), 10244. 10.3390/ijms231810244 36142147 PMC9499610

[B103] Wang L. Li P. Feng K. (2023a). EGCG adjuvant chemotherapy: current status and future perspectives. Eur. J. Med. Chem. 250, 115197. 10.1016/j.ejmech.2023.115197 36780831

[B104] Wang W. Ma P. Gao W. Lu P. Ding X. Chen J. (2022). Nrf2 knockout affected the ferroptosis signaling pathway against cisplatin-induced hair cell-like HEI-OC1 cell death. Oxidative Med. Cell. Longev. 2022, 2210733. 10.1155/2022/2210733 PMC9270153 35814275

[B105] Wang X. Zhou Y. Wang D. Wang Y. Zhou Z. Ma X. (2023b). Cisplatin-induced ototoxicity: from signaling network to therapeutic targets. Biomed. and Pharmacother. = Biomedecine and Pharmacother. 157, 114045. 10.1016/j.biopha.2022.114045 36455457

[B106] Wu W. Li Y. He J. Yang J. Liu Y. (2024). Resveratrol shields against cisplatin-induced ototoxicity through epigenetic lncRNA GAS5 modulation of miR-455-5p/PTEN pathway. Int. Immunopharmacol. 138, 112464. 10.1016/j.intimp.2024.112464 38917526

[B107] Xiang Y. Xu H. Chen H. Tang D. Huang Z. Zhang Y. (2023). Tea consumption and attenuation of biological aging: a longitudinal analysis from two cohort studies. Lancet regional health. West. Pac. 42, 100955. 10.1016/j.lanwpc.2023.100955 PMC10700389 38075587

[B108] Xie D. Liu G. Zhu G. Wu W. Ge S. (2004). (-)-Epigallocatechin-3-gallate protects cultured spiral ganglion cells from H2O2-induced oxidizing damage. Acta oto-laryngologica 124 (4), 464–470. 10.1080/00016480410018278 15224876

[B109] Xu B. Li J. Chen X. Kou M. (2022). Puerarin attenuates cisplatin-induced apoptosis of hair cells through the mitochondrial apoptotic pathway. Biochimica biophysica acta. Mol. cell Res. 1869 (4), 119208. 10.1016/j.bbamcr.2021.119208 35032475

[B110] Yazici Z. M. Meric A. Midi A. Arınc Y. V. Kahya V. Hafız G. (2012). Reduction of cisplatin ototoxicity in rats by oral administration of pomegranate extract. Eur. Fed. Oto-Rhino-Laryngological 269 (1), 45–52. 10.1007/s00405-011-1582-2 21442422

[B111] Yin Q. Shi G. Zhu L. (2023). Association between gut microbiota and sensorineural hearing loss: a Mendelian randomization study. Front. Microbiol. 14, 1230125. 10.3389/fmicb.2023.1230125 37915857 PMC10616596

[B112] Yu H. H. Jung S. Y. Shin M. K. Park R. So H. S. You Y. O. (2010). Pueraria thunbergiana inhibits cisplatin-induced damage of HEI-OC1 auditory cells through scavenging free radicals. Phytotherapy Res. PTR 24 (6), 834–839. 10.1002/ptr.3027 19957243

[B113] Yuan C. Zhang H. Sun C. Zhang K. (2023). Efficacy and safety of *Ginkgo biloba* extract as an adjuvant in the treatment of Chinese patients with sudden hearing loss: a meta-analysis. Pharm. Biol. 61 (1), 610–620. 10.1080/13880209.2023.2190782 36999358 PMC10071945

[B114] Yumusakhuylu A. C. Yazici M. Sari M. Binnetoglu A. Kosemihal E. Akdas F. (2012). Protective role of resveratrol against cisplatin induced ototoxicity in Guinea pigs. Int. J. Pediatr. otorhinolaryngology 76 (3), 404–408. 10.1016/j.ijporl.2011.12.021 22261612

[B115] Zhang X. Qian J. Wei B. Zhang B. (2025). Flavonoids as modulators of Nrf2 signaling pathway in alleviating cisplatin-induced organ toxicity. Yangtze Med. 9, 52–77. 10.4236/ym.2025.91006

[B116] Zhang Y. He Q. Dong J. Jia Z. Hao F. Shan C. (2016). Effects of epigallocatechin-3-gallate on proliferation and differentiation of mouse cochlear neural stem cells: involvement of PI3K/Akt signaling pathway. Eur. J. Pharm. Sci. 88, 267–273. 10.1016/j.ejps.2016.03.017 27012759

[B117] Zheng S. Liu C. Tang D. Zheng Z. Yan R. Wu C. (2022). The protective effect of rutin against the cisplatin-induced cochlear damage *in vitro* . Neurotoxicology 90, 102–111. 10.1016/j.neuro.2022.03.005 35304134

[B118] Zhou Y. Zheng J. Li Y. Xu D. P. Li S. Chen Y. M. (2016). Natural polyphenols for prevention and treatment of cancer. Nutrients 8 (8), 515. 10.3390/nu8080515 27556486 PMC4997428

